# Efficient Ionovoltaic Energy Harvesting via Water‐Induced p–n Junction in Reduced Graphene Oxide

**DOI:** 10.1002/advs.202404893

**Published:** 2024-08-05

**Authors:** Yong Hyun Cho, Minho Jin, Huding Jin, Junghyup Han, Seungyeon Yu, Lianghui Li, Youn Sang Kim

**Affiliations:** ^1^ Program in Nano Science and Technology Graduate School of Convergence Science and Technology Seoul National University Seoul 08826 Republic of Korea; ^2^ Institute of Chemical Processes Seoul National University Seoul 08826 Republic of Korea; ^3^ Department of Chemical & Biological Engineering College of Engineering Seoul National University Seoul 08826 Republic of Korea; ^4^ Advanced Institute of Convergence Technology Suwon‐si 16229 Republic of Korea

**Keywords:** carrier dynamics, electrode junction, energy harvesting, ionovoltaic effect, solid–liquid interface

## Abstract

Water motion‐induced energy harvesting has emerged as a prominent means of facilitating renewable electricity from the interaction between nanostructured materials and water over the past decade. Despite the growing interest, comprehension of the intricate solid–liquid interfacial phenomena related to solid state physics remains elusive and serves as a hindrance to enhancing energy harvesting efficiency up to the practical level. Herein, the study introduces the energy harvester by utilizing inversion on the majority charge carrier in graphene materials upon interaction with water molecules. Specifically, various metal electrode configurations are employed on reduced graphene oxide (rGO) to unravel its distinctive charge carriers that experience the inversion in semiconductor type upon water contact, and exploit this characteristic to leverage the efficacy of generated electricity. Through the strategic arrangement of the metal electrodes on rGO membrane, the open‐circuit voltage (*V*_oc_) and short‐circuit current (*I*_sc_) have exhibited a remarkable augmentation, reaching 1.05 V and 31.6 µA, respectively. The demonstration of effectively tailoring carrier dynamics via electrode configuration expands the practicality by achieving high power density and elucidating how the water‐induced carrier density modulation occurs in 2D nanomaterials.

## Graphical abstract

Formation of a p–n junction is observed across wet‐dry regions of reduced graphene oxide as water contact region experiences a type inversion from p‐ to n‐type. Asymmetric application of metal electrodes has been utilized to validate and leverage the inverted semiconductor type, hence amplifying electricity generation where the open‐circuit voltage (*V*_oc_) and short‐circuit current (*I*_sc_) have reached up to 1.05 V and 31.6 µA, respectively

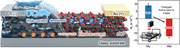

## 1 Introduction

The growing severity of global environmental issues such as water pollution, air quality degradation, and climate change attributed to the process of electricity generation underscores the imperative for scholarly deliberation on the implementation of alternative energy sources. In particular, a water motion‐induced energy harvester (WMEH) emerges as a relatively novel field of study yet bears remarkable potential.^[^ ^1^, ^2^, ^3^, ^4^ ^]^ WMEH not only addresses the environmental and stability challenges highlighted by conventional power sources but also draws immense advantage from the ubiquity nature of water on earth which covers over 70% of the surface. However, despite the promising attributes of the technology, WMEH has only recently surpassed a decade of academic exploration, leaving the clear elucidation of its driving principle yet to be conclusive. Numerous research endeavors, encompassing various interpretations of the energy conversion principles, are currently underway which are hydrovoltaic,^[^ ^5^, ^6^, ^7^ ^]^ triboelectric,^[^ ^8^, ^9^ ^]^ Coulombic‐drag,^[^ ^10^, ^11^ ^]^ pseudo‐streaming,^[^ ^12^, ^13^, ^14^ ^]^ and ionovoltaic,^[^ ^3^, ^15^, ^16^, ^17^, ^18^, ^19^ ^]^ all striving to expand the practicality and applicability of WMEH. Moreover, the predominant research heavily focuses on exploring diverse 2D‐based active materials to improve energy generation efficiency beyond current thresholds, such as graphene,^[^ ^5^, ^6^, ^20^ ^]^ metal oxides,^[^ ^21^, ^22^ ^]^ and composite materials.^[^ ^23^, ^24^ ^]^ However, regardless of the mechanistic approaches or material types employed, understanding the fundamental principles of the water‐induced interfacial phenomenon behind solid‐state physics remains of utmost importance in enhancing energy conversion efficiency. Still, this is largely neglected due to current analytical limitations concerning solid–liquid interfacial phenomena occurring at the nanoscopic region. Also, the water‐induced energy conversion mechanism primarily involves the electrostatic interaction of water molecules or ions that cause carrier density alterations within the solid material,^[^ ^12^, ^25^, ^26^, ^27^, ^28^, ^29^, ^30^, ^31^ ^]^ but the precise underlying mechanism has not been fully elucidated. Hence, it is crucial to accurately address water dynamics and corresponding behaviors of specific electrical properties in semiconductors including charge carrier dynamics along the solid surface.

Herein, an exposition of novel analytical approaches to water‐induced inversion layers in semiconductors and their respective mechanistic interpretations of energy generation are proposed. Specifically, we explore and develop a methodology to confirm the alterations in semiconducting properties via the perspective of charge carrier dynamics, resulting from ionovoltaic (ionic dynamics at the interface) effect at the reduced graphene oxide (rGO) surface. Ionovoltaics has surfaced as a convincing explanation for WMEH throughout multiple experimental validations, wherein the asymmetric electronic energy density by ionic charges within the liquid has been demonstrated to play a decisive role in electricity generation. The scientific pursuit has expanded beyond energy harvesting purposes to a broad spectrum of research domains centered on solid–liquid interfaces such as biomolecule and chemical sensors.^[^ ^32^, ^33^, ^34^ ^]^ To further explore these advancements, an in‐depth examination is conducted utilizing computational simulations and Hall effect measurement analyses to substantiate the unique traits demonstrated within the solid surface by charge carrier density alteration upon water exposure. Therefore, a comprehensive metal electrode configuration on rGO is designed to prove both the flow and density modulation of charge carriers, discerned via the analysis of the current‐voltage (*I‐* *V*) curve. By asymmetrically arranging the preferred metal electrode configuration on rGO, the open‐circuit voltage (*V*_oc_) and short‐circuit current (*I*_sc_) have increased up to 1.05 V and 31.6 µA. Through this remarkable electrical performance enhancement achieved by maneuvering charge carrier dynamics, we provide a step‐closer outlook of elucidating mechanistic intricacies associated with the relationship between energy generation and charge carrier movement, thus broadening the practicality of water motion‐driven energy harvesting systems.

## 2 Results and Discussion

**Figure 1:**
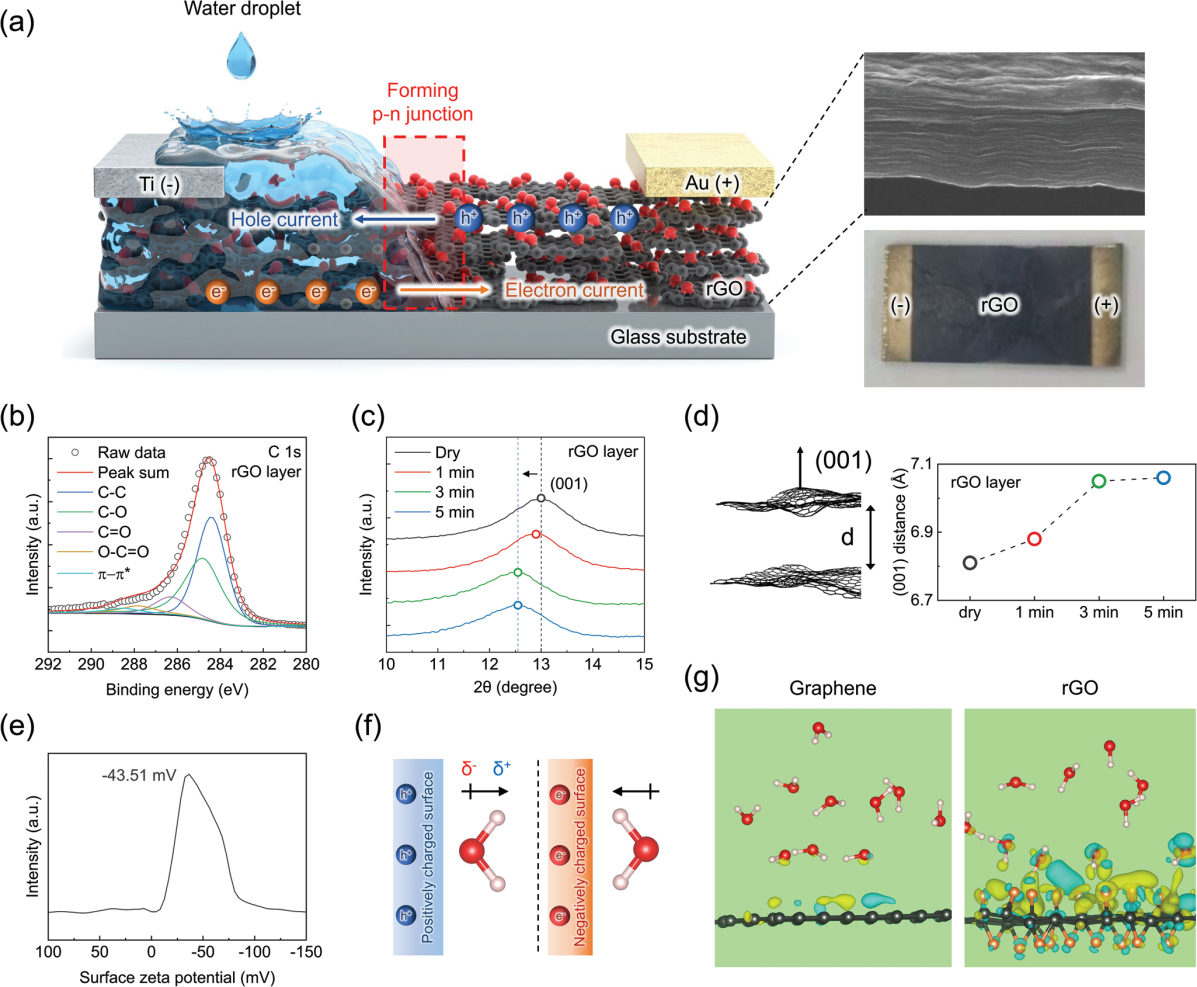
Structural properties of rGO and its characteristics upon water exposure. a) Schematic illustration of a rGO ionovoltaic energy harvester with metal electrodes deposited on each end for bolstering carrier dynamics by dropping water droplets. The multi‐layered structure of the rGO membrane obtained using cross‐sectional scanning electron microscopy (top right) and a picture of the device (bottom right). b) XPS spectrum shows the carbon‐oxygen composition and bonding states of rGO. c) XRD spectrum demonstrates that absorption of water over time leads to the interlayer expansion of the rGO membrane as indicated by the leftward shift of XRD peaks relative to its dry state. d) The gap distance gradually increases while water is being absorbed until it reaches a saturation point at ≈7.1 Å through the XRD data. e) Negative surface charge of −43.51 mV was confirmed by zeta potential measurement. f) Graphical presentation on how the orientation of the water molecule would be positioned along each of positively and negatively charged surfaces. g) Charge density distribution on pristine graphene and oxygen‐functionalized rGO in the presence of water molecules. Red: oxygen in water molecule, pink: hydrogen, black: carbon, orange: oxygen in epoxy group. Yellow and blue graphics indicate charge accumulation and depletion, respectively. isosurface: 0.0005 e Å^−3^.

**Figure** **1**a illustrates the absorption of water into the porous rGO ionovoltaic energy harvester with metal electrodes deposited at both ends of the device. Fabrication of devices with structures capable of eliciting sustained water movement is crucial for water‐induced energy harvesting systems since water movement is the driving force to charge carriers within 2D nanomaterials that generate electricity. Therefore, graphene oxide (GO) is thermally reduced to remove oxygen functional groups, preventing its intrinsic soluble and dispersing nature upon exposure to water or any other organic solvents.^[^ ^35^ ^]^ Pictures on the right side of Figure 1a show well‐arranged multilayer stacks of graphitic layers as captured by field emission scanning transmission electron microscope (FE‐SEM) and a picture of the device without any discernible structural deformation or detachment from poor affinity to the substrate. The fabricated partially reduced GO has various carbon‐oxygen bonds (C–O, C═O, O–C═O) that are well preserved even after thermal reduction,^[^ ^36^ ^]^ which is confirmed by X‐ray photoelectron spectroscopy (XPS) measurement, as shown in Figure 1b. Furthermore, annealing at a mild temperature of 120 °C enables the retention of a sufficient amount of oxygen groups, thereby facilitating water uptake, as shown via X‐ray diffraction (XRD) in Figure 1c, without any graphitic flake dissipation from the interaction with water which is common for GO. The contact angle of the rGO is shown in Figure S1 (Supporting Information), where a mild degree of annealing temperature applied to GO preserves an adequate level of oxygen groups, maintaining its hydrophilic characteristics with a contact angle below 90°. As the duration of water exposure continues, the XRD peak gradually shifts where the peak eventually reaches a saturation point at ≈5 min, showing that it no longer shifts. The shift correspondingly serves as an indication of the interlayer distance as depicted in Figure 1d, where water infiltrates through the porous rGO membrane expanding gaps between layers.^[^ ^37^ ^]^ The expanded van der Waals gap distance from water interaction is ≈0.28 Å and saturation is gradually reached. To assess the viability as a WMEH, it is crucial to initially confirm the semiconductor properties of the prepared material as the potential induction of charge density modulation at the solid–liquid interface occurs when water molecules interact with an intrinsic negative zeta potential surface (Figure 1e).^[^ ^5^, ^12^, ^27^, ^28^ ^]^ Also, while GO possesses insulating properties, making it unsuitable for energy harvesting applications, the reduction process transitions it to rGO which exhibits decreased resistance and a bandgap in the range of a semiconductor (Figure S2, Supporting Information). Hence, the interfacial effect from the water molecule interaction is first observed through density functional theory (DFT) calculation, a computational model demonstrating how ionovoltaic effect of water molecules induces charge attraction or depletion within the active material. The water molecule has zero net charge but there is a distribution of charge within the liquid from dipole moment between the atoms, where the oxygen atom has a slight negative charge and hydrogen atoms have a positive charge. The orientation of the molecule by which the atom comes near the solid surface can cause different charge density modulations along the interfacial region. Therefore, the intrinsic zeta potential and functional groups present on the graphitic surface that govern the orientation of the molecule are taken into consideration as a number of oxygen groups still remain even after the reduction process. In particular, a negatively charged surface would attract hydrogen atoms of a water molecule, whereas a positively charged surface would attract the oxygen atom (Figure 1f).^[^ ^38^ ^]^ In addition to intrinsic zeta potentials, the examination of functional groups on the surface is imperative due to their propensity to acquire positive or negative charge characteristics upon hydration and forming electrical double layer (EDL), thereby influencing molecular orientation. As shown in Figure 1g, the calculation results demonstrate that when considering pristine graphene with a negative surface, the induced charge modulation by water interaction that induces capacitive charging along the interface is relatively small. Contrary to the case of graphene, a significant enhancement in induced charge modulation is observed along the surface of rGO (Details of modeling crystal structure is provided in Figure S3, Supporting Information). This phenomenon can be attributed to the presence of oxygen functional groups which manifest a negative charge in addition to its negative zeta potential, thus fostering a stronger attraction to hydrogen atoms of water molecules. Due to the intrinsic defects in rGO, interaction between oxygen groups and water can accompany hydrogen bonding or redox reactions. These interactions can enhance the local carrier density and electric field in specific areas, thereby facilitating partial charge transfer.^[^ ^30^ ^]^ Consequently, the charge carriers (electrons in this case) within the rGO are more pronouncedly drawn toward the surface compared to the surface of pristine graphene, which hints at the possible formation of an inversion layer by water interaction. Various water molecule orientation‐dependent charge density distributions on rGO have been further demonstrated to confirm the influence of water molecule orientation on carrier density modulation in Figure S4 (Supporting Information). Drawing upon the captivating charge density modulation phenomenon observed via calculation results, we have formulated a strategy that effectively leverages the asymmetric carrier density across the wet‐dry regions of 2D materials by establishing the way for facilitating charge carrier dynamics and verifying their semiconductor types.

**Figure 2:**
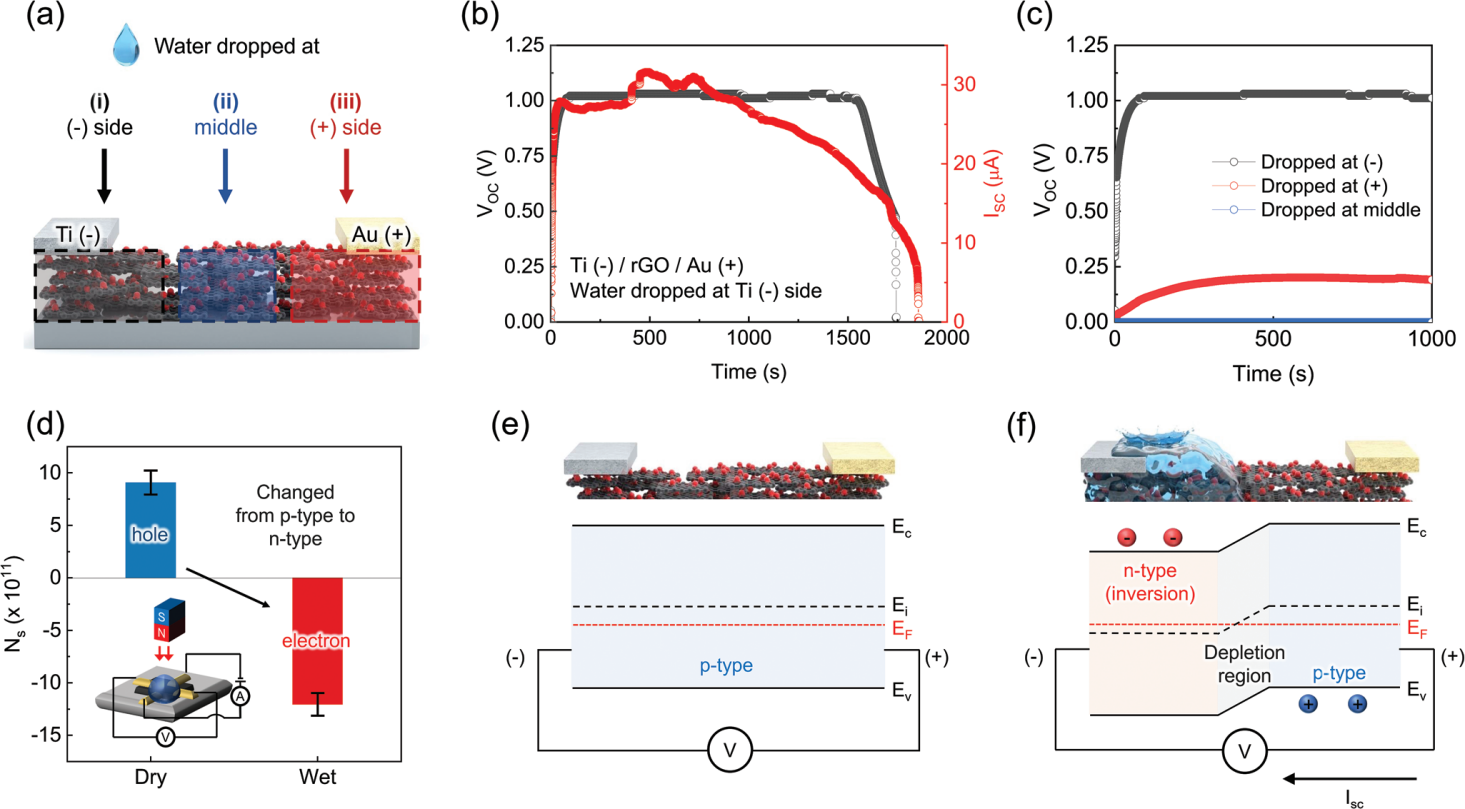
Electrical properties of rGO ionovoltaic energy harvester. a) Demonstration of water droplets dropping onto the (−), (+), and middle sections of the rGO component. b) Generated *V*_oc_ and *I*_sc_ at (−) side using 30 µL of a water droplet as a function of time. c) Corresponding voltage generated at each position. d) Hall measurement of dry state and wet state of rGO. Semiconductor‐type inversion occurs as electrons become dominant at the wet region. e) Band diagram of a dry state, p‐type rGO where the hole is the majority carrier. f) Band bending occurs as water interaction drives the wet‐state of rGO to undergo hole repulsion and thereby, n‐type inversion. Depletion region arises at the middle part of the interface boundary as the p–n junction is formed at wet‐dry regions of rGO.

The electrical properties of rGO as a WMEH device with metal electrode deposition are observed. Titanium (Ti) electrode and gold (Au) electrode are prepared at each end of the rGO device through thermal evaporation to probe how the charge carrier density and potential electrical type inversion are influenced by water interaction. While various metal electrodes are utilized, the influence of each deposited electrode on rGO and the rationale behind Ti(−)/Au(+) as the most effective among configurations will be discussed with band alignment theory in a later section. Importantly, Au is additionally deposited on top of Ti (Au‐capped Ti) without breaking the vacuum in a thermal evaporator as a passivation layer. The reason for this approach is to remove the risk of possible chemical reactions when Ti is exposed to air or water, as it is known to be susceptible to oxidation which may convert to TiO_2_.^[^ ^39^ ^]^ Perfectly covering the exposed surface of Ti with Au ensures the stability and integrity of the electrodes. Particularly, the preservation of the oxidation degree of the Au‐capped Ti electrode on rGO is assessed after exposure to water and air by scanning electron microscope‐energy dispersive X‐ray spectroscopy (SEM‐EDX) and X‐ray photoelectron spectroscopy (XPS) analyses (Figures S5 and S6, Supporting Information). No alteration in the proportion of oxygen components has been detected, alongside the absence of a shift in peaks in respective analyses, approving that the Au passivation layer is capable of effectively preventing Ti from unintended oxidation from air or water. Thereafter, the effect of the electrode‐deposited device on the electrical performance is investigated. A water droplet is dropped at (−) side, (+) side, and the middle section of the rGO (**Figure** **2**a). When a 30 µL of deionized (DI) water is dropped onto (−) side, voltage and current are continuously generated until water completely dries out. During the generation of electricity, *V*_oc_ and *I*_sc_ reach ≈1.05 V and 31.6 µA, respectively (Figure 2b). The performance of the device is further assessed by varying the volume of water droplets and conducting prolonged measurements to evaluate its stability in Figures S7 and S8 (Supporting Information). When increased amount of water is applied, the voltage remains unchanged but only the duration of electricity generation is extended. This indicates that the contact line (a demarcation line for the liquid area) has lasted longer. In the same manner, adjusting the humidity levels and temperature with a same amount of water reveals that a decrease in humidity and increase in temperature correspondingly reduced the duration of electricity generation (Figures S9 and S10, Supporting Information). This is also due to the more rapid evaporation of water within the nanoporous channels of rGO. The continuity of energy generation comes from sustaining asymmetric potential within the rGO device, as wet and dry regions seek to equilibrate their disparate energy states. An intriguing and notable observation is that when a water droplet is dropped for electricity generation, the device with asymmetrically deposited electrodes on each side generates more than twice the voltage and 20 times the current compared to that of the rGO without electrode deposition which generates *V*_oc_ and *I*_sc_ of 0.44 V and 1.56 µA, respectively (Figure S11, Supporting Information). The electricity generation measured at each (−) and (+) side without electrode deposition shows that the magnitude of voltage and current stays the same, but only the direction of the electricity generation is reversed. This signifies that the deposited electrodes along with the configuration affect charge carrier dynamics. Bare Ti without Au passivation layer is also measured for comparison (Figure S12, Supporting Information). While not significantly different, Au‐capped Ti generally demonstrates a slightly higher and more stable electrical energy generation. As previously discussed, this is likely attributed to the ability of the Au passivation layer that maintain Ti in a more stable state upon water interaction. The position where a water droplet is dropped also plays a crucial role in energy generation. It has been observed that when a water droplet is dropped on the middle section of the device, almost negligible energy is generated which serves as evidence that electricity generation arises from potential asymmetry within the device. When water is supplied on Au electrode where Au‐capped Ti electrode is deposited at the other side of rGO, continuous electrical energy is generated but has a significantly lower output compared to that of the Au‐capped Ti side (Figure 2c). Such output performance indicates that the electrode configuration has a great influence on the flow of charge carriers that contribute to energy output which could either greatly aid or hinder the carrier movement depending on the electrode contact types. Further insights are attained by supplying a water droplet at a distant position from the electrodes, confirming how carrier flow to the external circuit serves as an ionovoltaic energy generation source (Figure S13, Supporting Information). Additionally, the electrical energy is measured using NaCl solutions of different concentrations as shown in Figure S14 (Supporting Information). While there is no significant change, a slight increase in voltage to ≈1.2 V is observed as the concentration increased from 0.1 to 1 m. This is attributed to the increasing number of ions participating in nanofluidic conductance and amplifying asymmetric potential throughout rGO. The output signal tends to increase until the EDL is saturated. However, when using solutions with higher ion concentrations, such as 3 m, the screening effect of counter‐ions becomes dominant, leading to a decrease in the Debye length and output electricity. In a pursuit to gain a thorough elucidation of the internal carrier dynamics of the rGO device, we delved into how carrier density modulation unfolds from water interaction. Typically, graphene oxide is recognized for having p‐type characteristics with majority carriers of holes.^[^ ^40^ ^]^ This is the same trait observed in the prepared rGO device from multiple Hall effect measurements conducted under dry conditions, thus affirming the p‐type nature. However, noteworthy property alterations are observed following a water exposure, characterized by the formation of an inversion layer from p‐type to n‐type (Figure 2d). A reversal of electrical type could provide a compelling explanation behind the underlying reasons for the substantially enhanced electricity generation upon utilizing the asymmetric electrode configuration of Ti‐Au since the wet‐dry regions could have different preferred metal electrodes according to their electrical types. To delineate how carriers undergo type inversion during water interaction, electronic states of the energy band could best describe the carrier movement as shown in Figure 2e. Notice how p‐type rGO has a majority carrier of holes and fermi level (E_F_) under the intrinsic (E_i_) and close to valence bands (E_v_). There is no asymmetry in potential across the rGO device, so the carriers would not flow through the external circuit and generate electricity. On the other hand, in Figure 2f, water begins to infiltrate into the porous structure and divide rGO into wet and dry regions. The wet region is subject to type inversion as an electrostatic interaction of (+) dipole‐oriented water molecules pushes away holes and attracts electrons because of the negatively charged surface of rGO.^[^ ^38^ ^]^ The E_F_ of the wet region is now over the E_i_ and close to the conduction band (E_c_), making electrons the majority carriers. As the dry region remains as a p‐type material, it leads to the formation of a depletion region at the wet‐dry boundary along the n‐type wet region. Upon contact with rGO, the introduction of water initiates carrier density modulation in the wet region, resulting in the emergence of potential asymmetry within the rGO device. Consequently, carrier movement is driven as carriers would continuously flow to achieve a balance. During this process, as carriers traverse through the external circuit, electricity is generated (Figure 2f). The simultaneous water infiltration and natural evaporation at the middle section of the rGO membrane create water dynamics where the wet‐dry boundary continuously oscillates at a microscopic level.^[^ ^3^ ^]^ This phenomenon suggests that the carrier concentrations in the wet‐dry regions experience repeated fluctuations due to the movement of the wet‐dry boundary which is the driving force for carrier dynamics and sustained energy generation.

**Figure 3:**
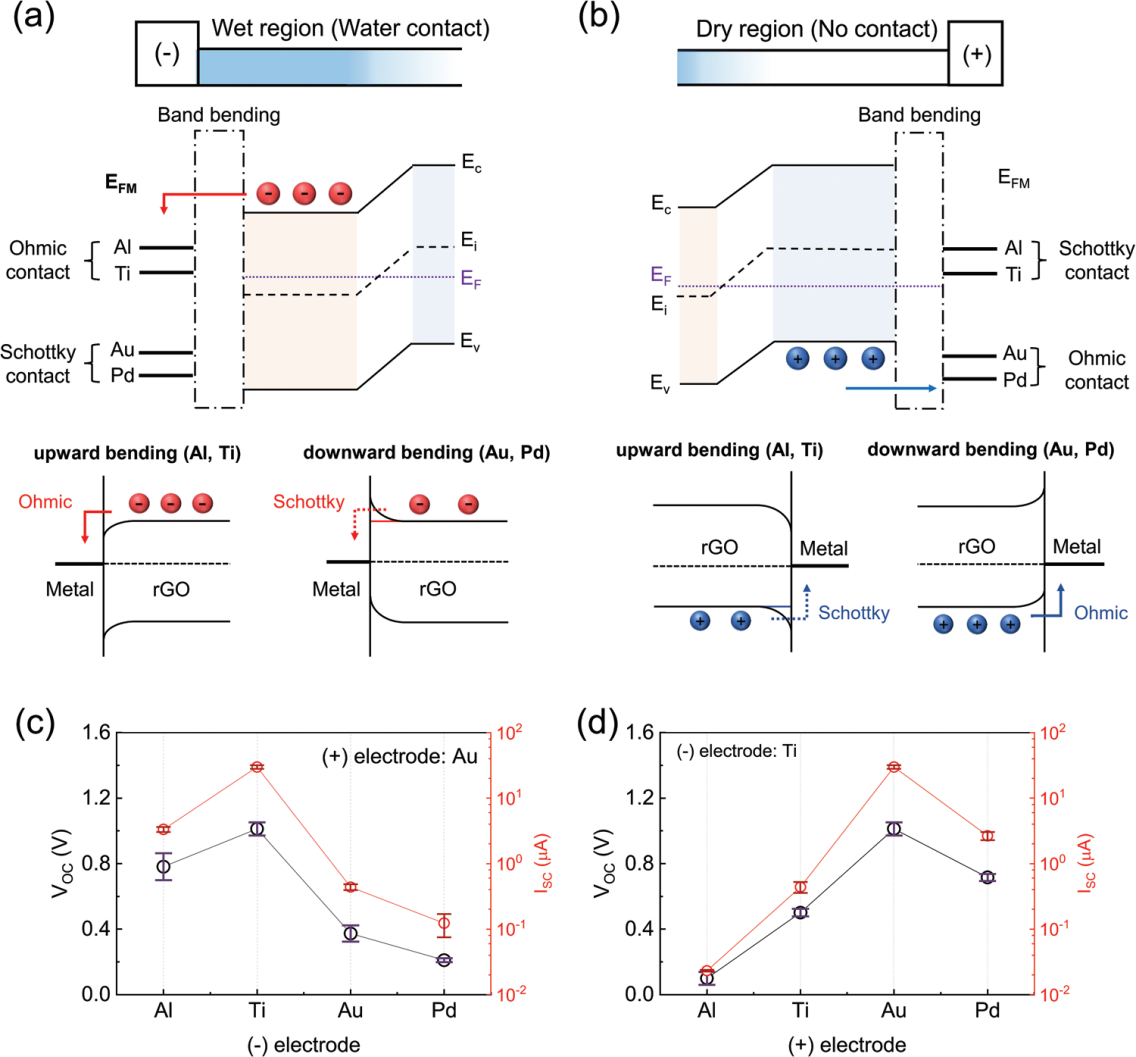
a,b) Band alignment and carrier dynamics of various metal electrodes effect (a) on the wet region with electrons as major charge carriers. b) on dry region with holes as major charge carriers. c,d) *V*_oc_ and *I*_sc_ for each electrode configuration c) varied electrodes deposited on the (−) side whereas (+) electrode is fixed with Au. d) varied electrodes deposited on the (+) side whereas (−) electrodes are fixed with Ti.

To further explore the effects of the semiconductor‐type inversion phenomenon with electrode configuration, we conducted investigations into the electrical characteristics by selecting various metal electrodes based on work functions and their ohmic/schottky contact type with rGO.^[^ ^41^ ^]^ Ohmic contact electrode facilitates conducive carrier movement forming a pathway that charge carriers can readily flow, thus aiding in electricity generation. On the other hand, schottky contact forms a potential barrier in the pathway to an external circuit, allowing only selective carriers to flow which results in significantly reduced electrical energy output. **Figure** **3**a describes how carriers on the wet region flow depending on each of the deposited electrodes. Aluminum (Al) and Ti are schottky contact electrodes with dry‐state rGO as their work function values are 4.28 and 4.33 eV, respectively, lower than that of rGO at 4.64 eV. Palladium (Pd) and Au are ohmic contact electrodes with rGO with higher work function values at 5.12 and 5.1 eV, respectively (Figure S15, Supporting Information). Transmission line model (TLM) analysis is conducted to assess the quality of electrode deposition onto the rGO surface by determining whether there are significant variations in contact resistance among the electrodes, potentially influencing carrier mobility within the active material (Figure S16, Supporting Information).^[^ ^42^ ^]^ As there were no substantial differences in contact resistance among the electrodes, the correlation of electrode contact types to electricity generation is examined as they induce ionovoltaic‐dependent energy band bending at the electrode‐rGO junction. In the case of the type inversion by water interaction, electrons would act as major carriers on the wet region and Ti and Al would convert its contact type from schottky to ohmic according to n‐type rGO (Figure 3a, bottom left illustration). This behavior indicates that placing initial schottky contact with Ti and Al electrodes at the part where water makes direct contact should generate enhanced energy output. Conversely, initial ohmic contact with Au and Pd electrodes now act as schottky contact electrodes upon contact with water (Figure 3a, bottom right illustration). Therefore, Au and Pd electrodes are considered less suitable for the wet region than the Ti and Al. Figure 3b illustrates the carrier dynamics of a dry‐state rGO, where the electrodes maintain the contact type initially formed with rGO. As water makes contact on the opposite (−) side, carrier density on the dry region may adjust as carriers strive to achieve balance across the rGO device. However, since there is no direct contact with water, a drastic semiconductor‐type alteration such as n‐type inversion does not occur. To provide solid evidence supporting the characterization change of rGO, the water‐induced electrical performance of each electrode configuration is measured three times for both voltage and current. Figure 3c depicts the graph where the Au electrode is fixed in the dry (+) region where water does not make contact and electrode variations are applied in the wet region (Details are presented in Figure S17, Supporting Information). Without electrical type inversion in rGO membrane, the optimal electrode configuration on rGO would be having either Au or Pd electrodes on both sides since they exhibit ohmic contacts. However, the actual electricity generation results show quite the opposite outcome. The Ti and Al electrodes on the wet (−) region generate significantly more electrical energy than Au and Pd, when the dry (+) region is fixed with Au, which suggests that carrier density modulation from the electrostatic interactions by water molecules has resulted in n‐type inversion (Figure 3c). This indicates that the Ti and Al that once formed schottky contact with rGO would function as ohmic contact electrodes, providing improved assistance with the carrier flow. Positioning Au or Pd on the wet (−) region establishes schottky contact where the electrode creates a barrier that prevents charge carriers from flowing to an external circuit. Therefore, Au(−)/Au(+) and Pd(−)/Au(+), schottky(−)‐ohmic(+) contact electrodes exhibit relatively decreased energy output and are considered less favorable configurations since only one pathway is open. Figure 3d shows electrical outputs with a Ti electrode fixed on the (−) side and varied electrodes on the (+) side. The (−) side of the rGO undergoes n‐type inversion upon interaction with water, consequently transitioning Ti electrode to serve as an ohmic contact electrode. Both Ti(−)/Au(+) and Ti(−)/Pd(+) configurations establish ohmic‐ohmic contacts, leading to the highest electricity generation. Every electrical measurement of electrode configuration is also provided (Figure S17 and Table S1, Supporting Information), as well as output performance comparison which shows a remarkable solid–liquid interfacial power output utilizing DI water (Table S2, Supporting Information). The deposition of asymmetric electrodes at both ends of the device according to their electronic energy states will serve as a valuable guide for understanding solid–liquid interfacial phenomena in 2D nanomaterial semiconductor properties.

**Figure 4:**
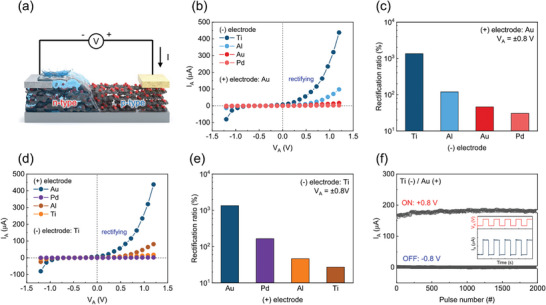
a) Graphical illustration of the current passing through the wet‐dry divided regions of rGO device while voltage is being applied. b) *I*–*V* curve analysis on different (−) electrodes when the (+) electrode is fixed with Au over the *V*_A_ range spanning from −1.2 to 1.2 V. c) Corresponding rectification ratios for the assessment of the diode characteristics at ±0.8 V upon water contact. d) *I*–*V* curve analysis on different (+) electrodes when (−) electrode is fixed with Ti over the voltage range spanning from −1.2 to 1.2 V. e) Corresponding rectification ratios at ±0.8 V. f) Transient response in Ti(−)/rGO/Au(+) diode to verify stable on/off function over time.

In the process of utilizing water to generate electrical energy, exploring the *I*–*V* curve could offer insights into how the current (*I*_A_) changes with different applied voltage (*V*_A_) levels. This approach not only could enhance the current state of shortcoming understanding of the liquid‐bound electronic states of 2D nanomaterials, but also could provide an additional experimental clue on n‐type inversion phenomena by showing diode characteristics across the wet‐dry regions of rGO.^[^ ^43^ ^]^ Here, as mentioned before, the water contact region experiences a type inversion from p‐type to n‐type, resulting in the formation of a p–n junction at the interface of the water contact line and the adjacent dry region (Figure S18, Supporting Information). **Figure** **4**a illustrates the experimental setup designed to measure the *I*–*V* curve. Similar to measuring voltage and current, water is dropped at the boundary of the deposited electrode and the active material to avoid direct contact with water and the probe tip. The *V*_A_ range is set between −1.2 and 1.2 V to preclude any potential induction of electrolysis reactions in the liquid medium.^[^ ^44^ ^]^ As shown in Figure 4b, *I*–*V* curves are acquired for each electrode configuration with the same combination employed in the preceding electrical measurements where the (+) electrode is fixed with Au. A recognizable observation here is that while there exists some variance in curve intensity when each of all electrodes is utilized, rectification behaviors are evident in the Al and Ti electrodes at the (−) side. Furthermore, it is noteworthy that electrode configurations such as Au(−)/Au(+), anticipated to be ohmic‐ohmic contact with dry‐state rGO, would not have manifested rectification behavior without water‐induced type inversion. Nonetheless, the presence of rectification behavior across all electrode combinations, including Au‐Au electrode configuration, unequivocally indicates evidence of n‐type inversion upon water contact with rGO. The intensity of the curves observed in the graph corresponds closely to the outcomes obtained from *V*_A_ and *I*_A_ measurements. Here, the breakdown is observed because of the tunneling of each charge carrier below the *V*_A_ of −0.8 V. This breakdown becomes more pronounced as the rectification ratio increases, indicating that the change in the fermi level is effectively observed when water droplets are dropped.^[^ ^45^ ^]^ When the Au is fixed as the (+) electrode, the sequence of *V*_oc_ and *I*_sc_ observed for the (−) electrode follows the order Ti > Al > Au > Pd. Remarkably, this sequence is also reflected in the intensity of rectification behavior observed across the configurations in Figure 4c. Upon closer examination of the rectification ratio at ±0.8 V, it becomes apparent that the disparity in electrical performance of *V*_oc_ and *I*_sc_ and rectification ratio across each electrode configuration exhibits a similar level of variation. This implies that as the rectification ratio becomes more pronounced by the external *V*_A_, a greater quantity of charge carriers flows, expressed as *V*_oc_ and *I*_sc_, from each wet and dry region to the external circuit in the energy harvester. By inspecting the effect of electrode deposition, confirmation of water‐induced semiconductor‐type inversion is attainable. Moreover, in the context of WMEH utilizing 2D materials, it is clear that employing suitable electrodes following the intrinsic work function and semiconductor type is capable of augmenting the volume of carrier flow and hence, generating enhanced electrical energy. A similar degree of change can also be observed when the (−) electrode is fixed as Ti, while various electrodes are deposited as the (+) electrode (Figure 4d). The observed sequence of rectification behavior follows Au > Pd > Al > Ti as a (+) electrode. This pattern correlates with the same trend in water‐induced energy harvesting efficiency levels as well. Au and Pd electrodes on the (+) side excel in generating substantial electrical energy which is attributed to the formation of ohmic contacts with a dry region in rGO membrane, as evident from the intensified curve formations in the *I*–*V* curve. As demonstrated in Figure 4e, the rectification ratio is higher for electrode configurations with ohmic‐ohmic contacts compared to those with ohmic‐schottky contact configurations. Similarly, the amount of electrical energy generated when water is dropped also bears a strong resemblance to the rectification ratio trends observed from each configuration. Finally, to ascertain the stable operation of the rGO diode, it was subjected to repeated on/off cycles over a period of time. As evident in Figure 4f, the diode exhibited a clear on/off function for 1000 cycles, demonstrating its capability for stable performance. Therefore, by correlating the electrical current flowing through rGO membrane with the applied voltage, insights into the ionovoltaic effect of electronic states were attainable which has provided a decisive clue for examining semiconductor property alterations within the active material from water interaction. Additionally, analyzing the rectification ratio in diode characteristics has served as a guide that provides analytical references for identifying how the electrode‐semiconductor junction effect governs charge carrier flows and contributes to effectively generating electricity when utilizing 2D nanomaterials as the WMEH.

## 3 Conclusion

In conclusion, the water contact‐driven interfacial effect is utilized to induce potential asymmetry across wet‐dry regions, thereby facilitating carrier dynamics and effective ionovoltaic energy harvesting. The examination of semiconducting property alterations was conducted due to the direct impact on carrier mobility. Through Hall effect measurement, we observed changes in carrier density within the wet region and subsequent electrical type inversion, which were further corroborated by electrode configuration and *I*–*V* curve analyses on rGO. Specifically, the asymmetric application of metal electrodes has been utilized to validate and leverage the inverted semiconductor type at the wet region, hence amplifying carrier dynamics and electricity generation where *V*_oc_ and *I*_sc_ have reached up to 1.05 V and 31.6 µA, respectively. Additionally, an *I‐*‐*V* curve analysis conducted over deposited metal electrodes on rGO verifies the n‐type inversion as a p–n junction is formed at the wet‐dry boundary. Overall, there is a concerted effort to explore various materials to surpass current thresholds in harnessing electrical energy, yet the fundamental comprehension of semiconducting behavior from water interaction has been largely neglected. Through novel analyses of the solid–liquid interfacial phenomena and strategic electrode deposition, the commercial viability of WMEH technology has been notably propelled by providing a guide to adeptly maneuver carrier dynamics of 2D materials, thereby boosting electricity generation efficiency and broadening their applicability.

## 4 Experimental Section

### Preparation of rGO Energy Harvesters

GO solution at a concentration of 20 g L^−1^ (Grapheneall) was initially diluted down to 12 g L^−1^ using DI water and 1.5 mL of the diluted GO solution was drop‐casted onto a glass substrate (1 × 2 cm^2^). After drying the solution in an ambient environment, GO suspension was taken into an oven for a thermal reduction process. Annealing was carried out very carefully as abrupt temperature increases would strip away excessive amounts of oxygen functional groups that can cause structural deformation from breaking carbon–oxygen bonds. Hence, meticulous handling was necessary for the reduction process to maintain the integrity and adhesion of the resulting film. The stepwise annealing process involved thermal reduction at specific temperatures and durations: 60 °C for 3 h, followed by 90 °C for another 3 h, and at 120 °C for a final 6 h period. This gradual annealing ensured that the rGO remained firmly adhered to the glass substrate even after reduction without any cracks. A metal mask was then used to expose the part of rGO device for depositing the desired metal electrode with a thickness of ≈200 nm using metal sources in a thermal evaporator. While utilizing various metal sources for electrode deposition, the internal temperature remained at a maximum of ≈40 °C ensuring that any unintended reduction is avoided. The deposited electrode had a size of 0.5 cm each at both ends of the rGO energy harvester.

### Characterization of rGO

XPS and UPS were measured with a photoelectron spectrometer (NEXSA, ThermoFisher). SEM‐EDX was measured with a field emission scanning electron microscope (S‐4800 D8, Hitachi). XRD with an X‐ray diffractometer (Discover, Bruker). Hall measurement was conducted to investigate the semiconductor type and majority carrier density by Hall effect measurement system (HL5500, Nanometrics). The bandgap of rGO films was measured from 300 to 800 nm using ultraviolet–visible (UV–vis) spectroscopy (Lambda 35, PerkinElmer).

### Electrical Measurements

The electrode clip was clipped on each of the deposited metal electrodes and a water droplet was dropped onto the interfacial boundary region between the deposited metal electrode and rGO. This was to prevent water from directly contacting the clip of the electrode wire. The voltage and current were measured using a nanovoltmeter (Keithley 2182A) and a picoammeter (Kethley 6485), respectively.

### *I–V* Curve Analysis

A water droplet was dropped onto the same interfacial region to measure electrical properties. The tip was gently placed onto the electrode and the voltage range was set from −1.2 V to 1.2 V so as to prevent a potential water hydrolysis reaction. The wet region of rGO was connected to the negative port of a Keysight 2902B source/measure unit while the dry region of rGO was connected to the positive port.

## Conflict of Interest

The authors declare no conflict of interest.

## Supporting information

Supporting Information

## Data Availability

The data that support the findings of this study are available from the corresponding author upon reasonable request.
