# Supplementary material for: Efficient Ionovoltaic Energy Harvesting via Water‐Induced p–n Junction in Reduced Graphene Oxide
Source: Adv Sci (Weinh). 2024 Aug 5;11(38):2404893. doi: 10.1002/advs.202404893 (PMC11481184; doi:10.1002/advs.202404893)
Supplement: Supplementary file 1 — Supporting Information [file ADVS-11-2404893-s001.docx]

Supporting information

**Efficient Ionovoltaic Energy Harvesting via Water-Induced p-n Junction in Reduced Graphene Oxide**

*Yong Hyun Cho, Minho Jin, Huding Jin, Junghyup Han, Seungyeon Yu, Lianghui Li, and Youn Sang Kim**

Y. H. Cho, M. Jin, Y. S. Kim

Program in Nano Science and Technology, Graduate School of Convergence Science and Technology, Seoul National University, Seoul 08826, Republic of Korea

H. Jin, Y. S. Kim

Institute of Chemical Processes, Seoul National University, Seoul 08826, Republic of Korea

H. Jin, J. Han, S. Yu, L. Li, Y. S. Kim

Department of Chemical & Biological Engineering, College of Engineering, Seoul National University, Seoul 08826, Republic of Korea

Y. S. Kim

Advanced Institute of Convergence Technology, Suwon-si, 16229, Republic of Korea

**Supporting Note 1. Method for DFT calculation and crystal structure design**

First principle calculations were performed using Quantum Espresso V7.1. within the density functional theory (DFT) calculation, incorporating the pseudopotential of each atom through projector augmented wave (PAW). The exchange-correlation functional was handled using the generalized gradient approximation (GGA), following the parametrization scheme of Perdew, Burke, and Ernzerhof (PBE). In the process of converging scf energy, calculations within the crystal were conducted using k-points of 256, and a convergence threshold was set at 2 × 10^-7^ Ry (about 2.721 × 10^-6^ eV). The rGO structure cell was configured by periodically bonding epoxide (–O–) to graphene for the highest energetical stability.^[1]^ As shown in Figure 1g, 10 water molecules were randomly distributed on the surface of graphene and rGO within the supercell to calculate charge density. Subsequently, for structural optimization, the positions and orientations of water molecules that yielded the highest structural stability were determined, along with the consequent distortion in the structure of graphene and rGO. Here, to describe the physisorption on the surface by water molecules, we incorporated the Grimme-D3 van der Waals correction during the scf energy harvesting process. The charge density plot derived from these energy calculations represents the changes in electron charge density. The electron density distribution across the x, y, and z axes was visualized through a 3D plot.

**Supporting Note 2. Schottky contact in metal-semiconductor junction**

The electron flow through schottky contact is explained by thermionic emission at the metal-semiconductor junction as follows,

$I_{S\to M}=AKT^{2}e^{\frac{-q(\varphi_{B}-V_{b})}{kT}}$ (S1)

$\varphi_{B}=\left\{ \begin{aligned} E_{Fm}-E_{c} \left( E_{Fm}>E_{c} \right) when the semiconductor is n-type \\ E_{v}-E_{Fm} \left( E_{v}>E_{Fm} \right) when the semiconductor is p-type \end{aligned} \right.$ (S2)

$K=\frac{4\pi qm_{n}k^{2}}{h^{3}}$ (S3)

where $I_{S\to M}$ is the emission current from the semiconductor to metal, $A$ is the contact area, $T$ is the temperature, $q$ is the Coulomb constant, $\varphi_{B}$ is the barrier potential, $V_{b}$ is applied effective voltage, k is the Boltzmann constant, $E_{Fm}$ is the work function of metal, $E_{c}$ is the conduction band of semiconductor, $E_{v}$ is the valence band of semiconductor, $m_{n}$ is the mass of electron, and $h$ is the Planck constant. Through this equation, it can be observed that $I_{S\to M}$ decreases exponentially as $\varphi_{B}$ increases even when the $V_{b}$ is fixed. Therefore, as the difference between $E_{Fm}$ and $E_{c}$ (n-type) or $E_{v}$ (p-type) increases, the obtained I_sc_ dramatically decreases when the metal and semiconductor form schottky contact in their junction.

**
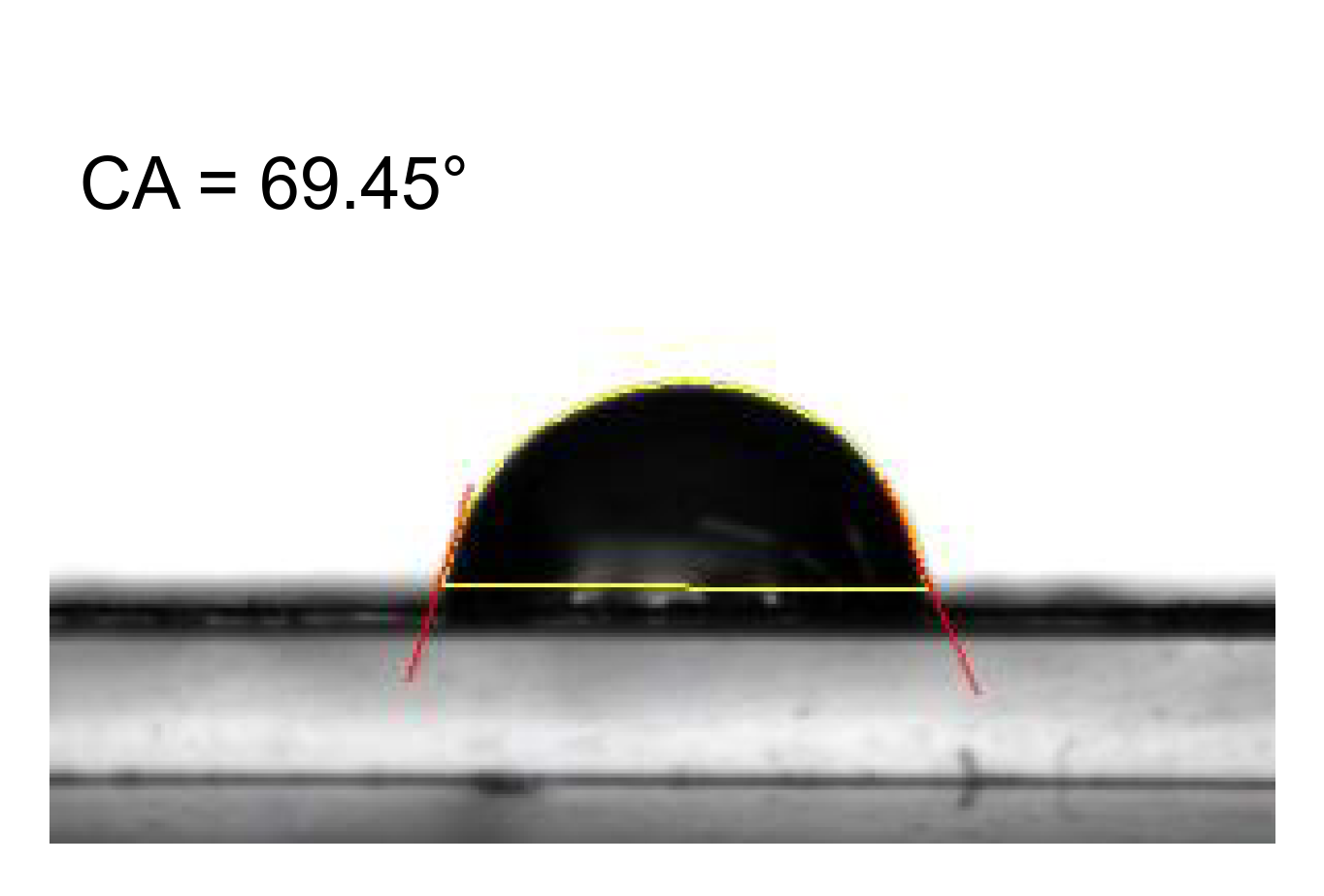
**

**Figure S1.** The water contact angle of the rGO is at 69.45°. A moderate degree of annealing temperature sustains sufficient oxygen groups to facilitate water absorption.


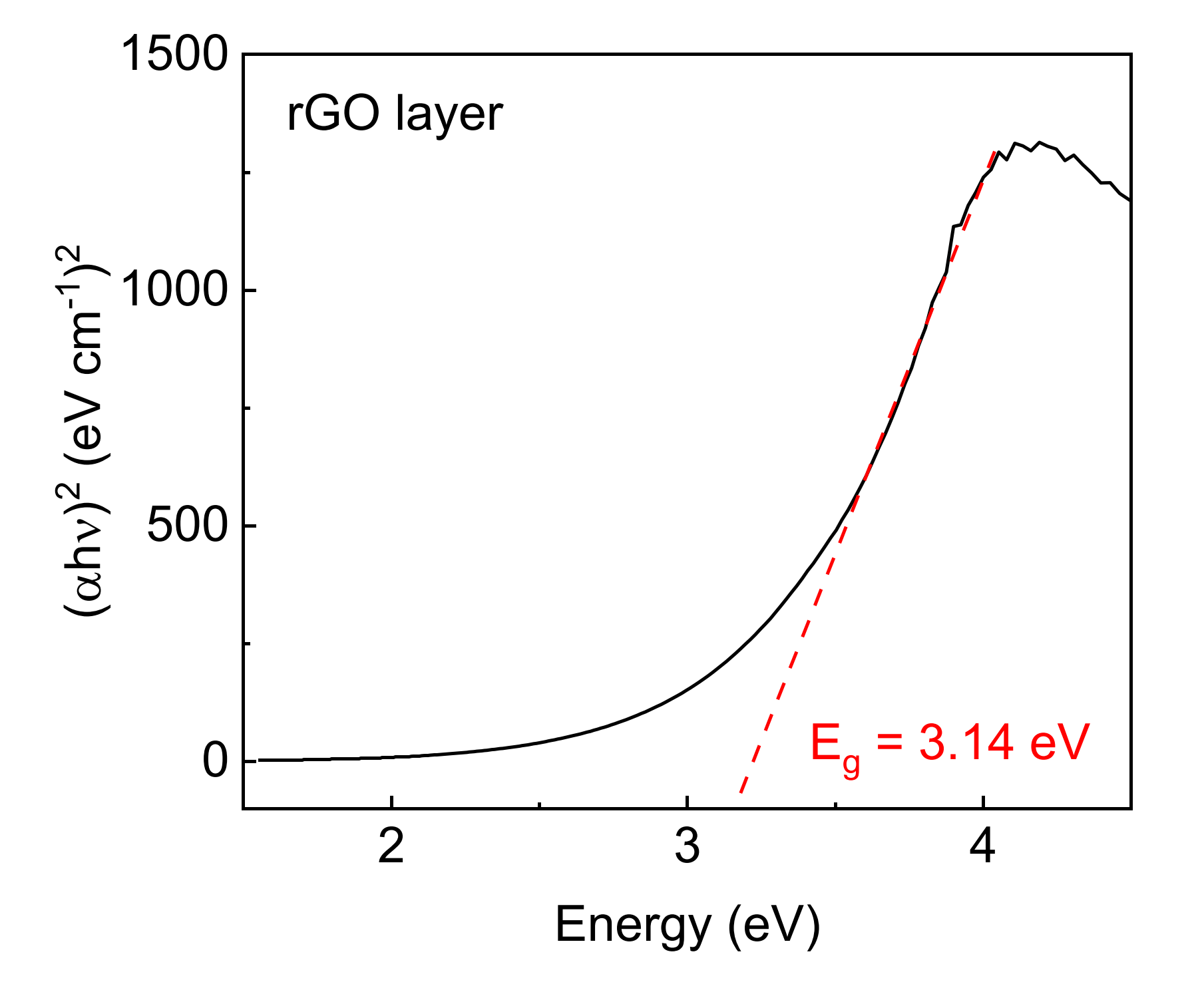


**Figure S2.** The bandgap of rGO is measured with UV-Vis analysis. Graphene is a zero-gap material that conducts electricity like a metal, which is unsuitable for utilization for energy harvesting purposes. On the other hand, rGO used in this study has a bandgap of 3.14 eV that could act as a semiconductor.

**Figure S3.** Modeling rGO crystal structure for first principle calculation. black: carbon, orange: oxygen. The most energetically stable structure of graphene oxide has a ratio of carbon 2 : oxygen 1 (see the atomic ratio of actual rGO in Figure S5) with periodically alternating epoxy groups above and below the graphene lattice. Here, while actual reduced graphene oxide contains other functional groups such as carboxyl or hydroxyl groups, and defects exist between carbon atoms, these aspects were not considered for the sake of simplicity in calculation.
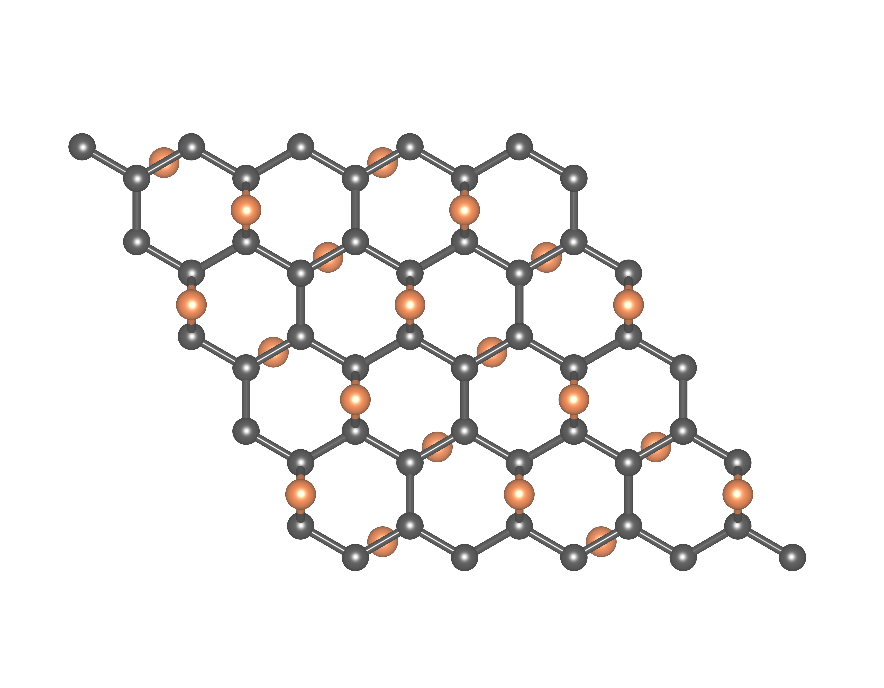


**
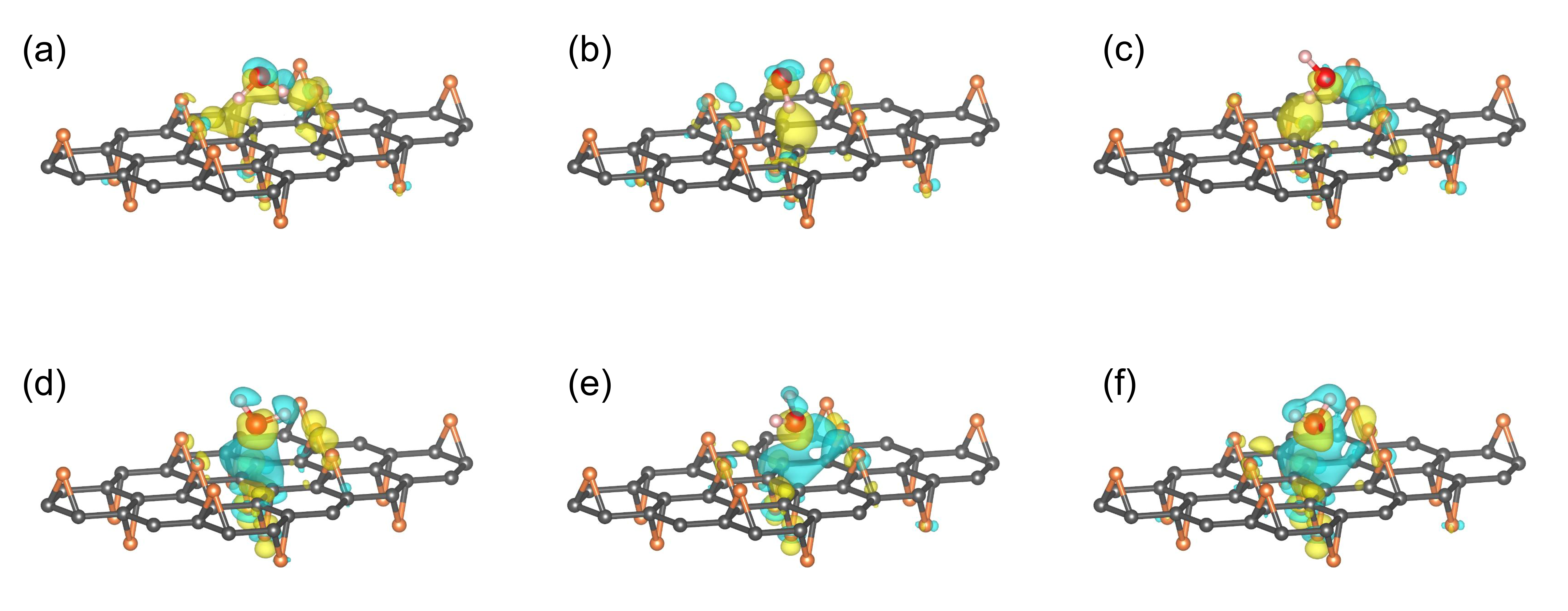
**

**Figure S4.** Water molecule orientation-dependent charge density distribution. Red: oxygen in water molecule, pink: hydrogen, black: carbon, orange: oxygen in epoxy group. Yellow and blue graphics indicate charge accumulation and depletion, respectively. isosurface: 0.001 e Å^-3^. a-c) The three illustrations at the top have hydrogen atoms facing toward the surface of rGO but with slightly different orientations which results in varying degrees of magnitude on the charge accumulation.^[2]^ Hydrogen atoms facing rGO are expected to occur as the negative zeta potential of rGO and oxygen functionalized surface will attract the positive charges under hydration. They attract opposite charge carriers (electrons) within the active material, leading to the accumulation of charges at the interface. d-f) For the three illustrations at the bottom where the oxygen atom of the water molecule has come near the rGO surface, positive charge carriers (holes) would be attracted to the interface. However, as the oxygen part of a water molecule is deliberately drawn to the negative surface, charge depletion is shown at the interface region. Given the substantial charge density variations resulting from the interaction between molecular orientation and solid surfaces, it is crucial to thoroughly comprehend the intrinsic characteristics of the active material and consider its interaction with liquid molecules.

**
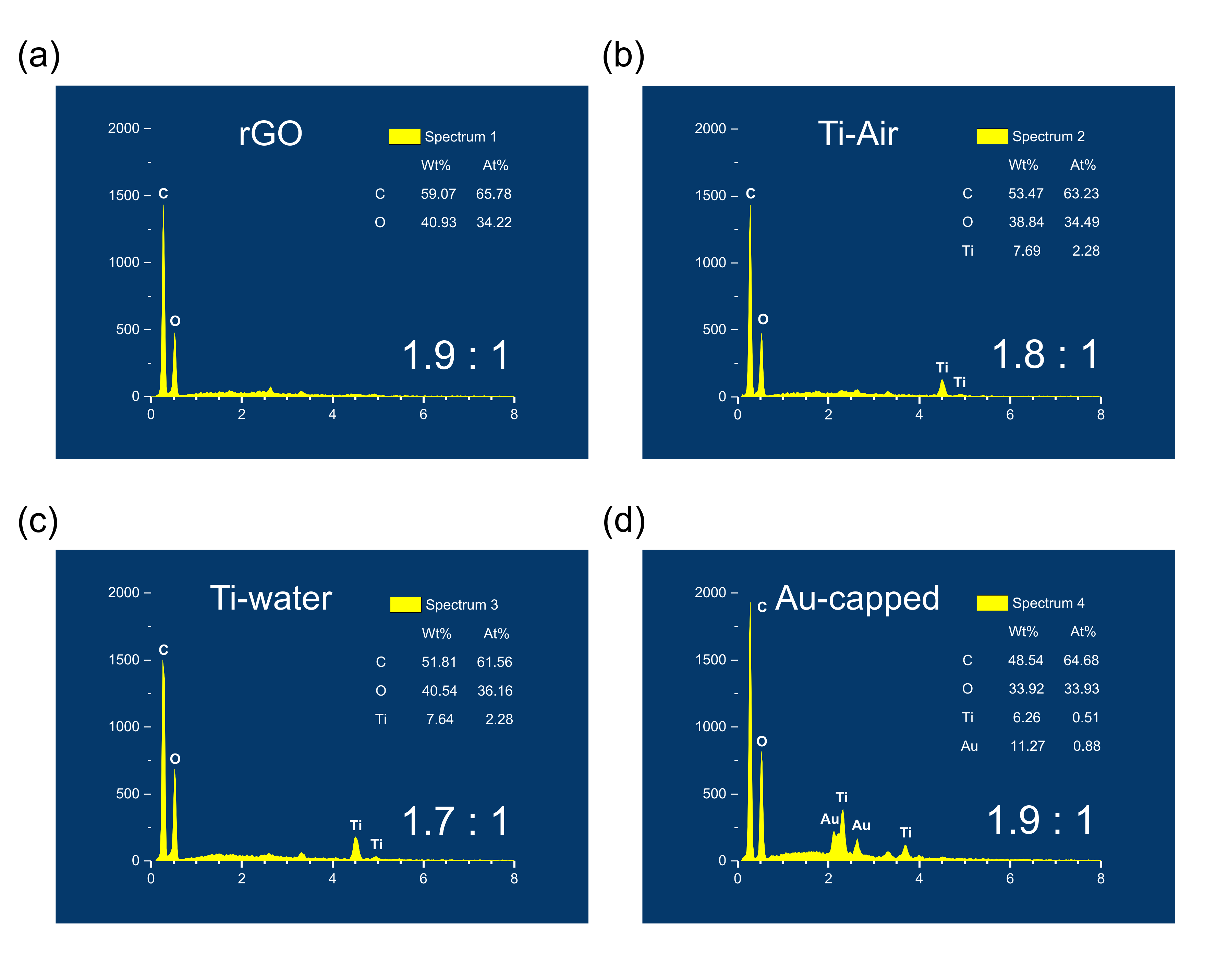
Figure S5.** The carbon to oxygen ratios of the rGO and Ti electrode-deposited devices are compared via SEM-EDX analysis. a) rGO indicates pristine rGO membrane without electrode deposition. b-c) Ti-air and Ti-water denote the Ti-deposited rGO that has undergone exposure to ambient air and water for 10 mins, respectively. d) Au-capped denotes the Au-capped Ti deposited rGO that has undergone exposure to water for 10 mins. Weight percent (wt%) and atomic percent (at%) of the element composition is achieved for the comparison. A water droplet is used to completely wet the electrode-deposited region and just before the analysis, water is removed. For the pristine rGO, the carbon to oxygen (C/O) ratio was about 1.9:1. Following the deposition of Ti onto rGO and subsequent exposure to ambient air for 10 mins, a subtle elevation in oxygen composition has occurred, resulting in a C/O ratio of 1.8:1, Upon exposing the Ti-deposited device to water for 10 mins, a C/O ratio of 1.7:1 was observed. While the difference in oxygen proportion is not significant from that of the pristine rGO, a slight increase in oxygen proportion is evident, indicating possible oxidation of Ti to TiO_2_ has occurred from exposure to air and water. Conversely, when Au-capped Ti electrodes deposited on rGO are subject to the same 10 mins of water exposure, the resulting C/O ratio exhibited no alteration compared to pristine rGO.


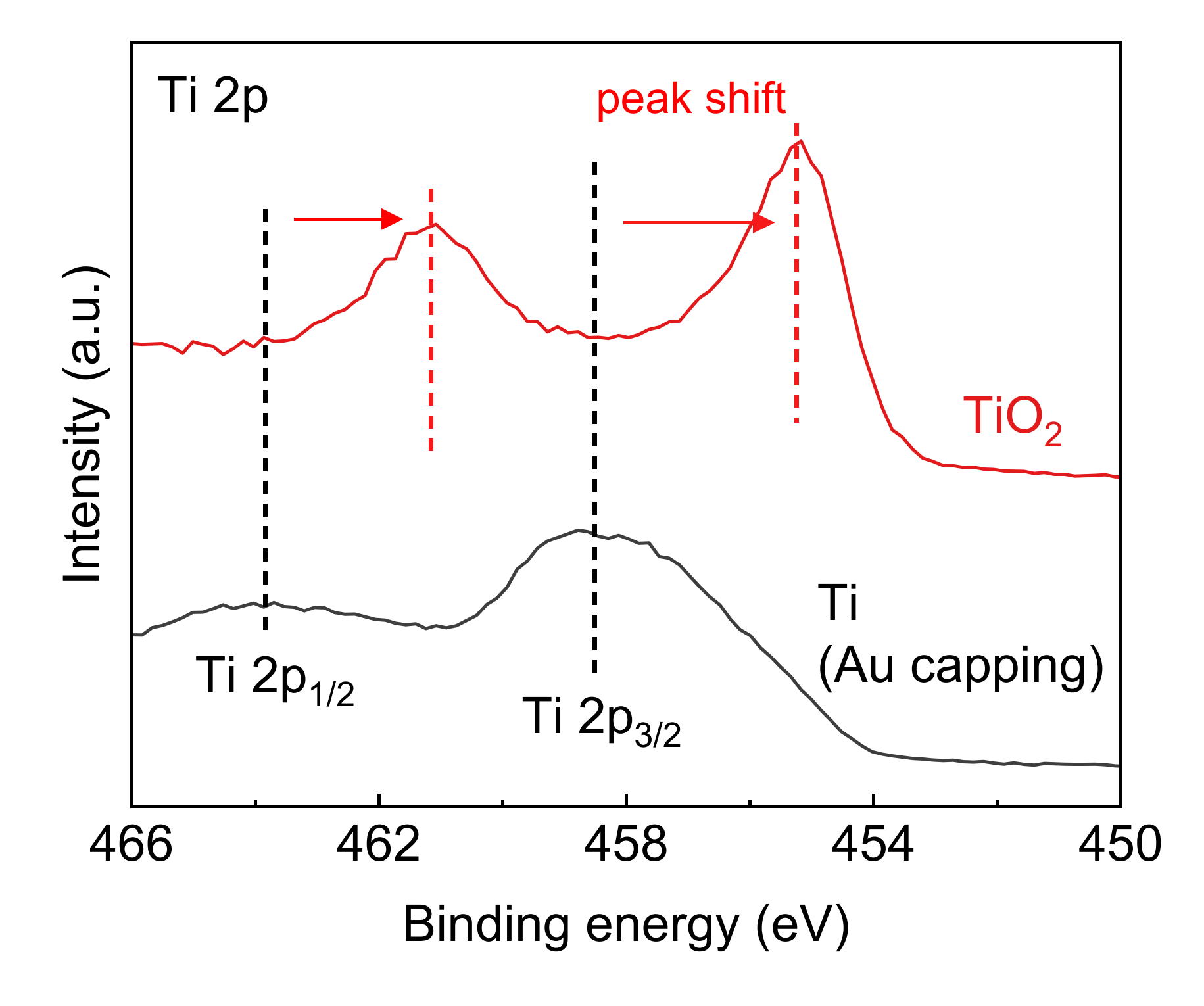


**Figure S6.** The oxidation degree of the Au-capped Ti electrode under water exposure is measured by XPS. Ti electrode tends to be oxidized when water or air comes into contact. The red curve in the graph is what the potential chemical reaction of having oxidized Ti would exhibit, shifting the curve toward the right.^[3]^ However, even after direct water interaction for 10 mins, the Au-capped Ti electrode does not shift as oxidation is perfectly prevented by the Au passivation outer layer.


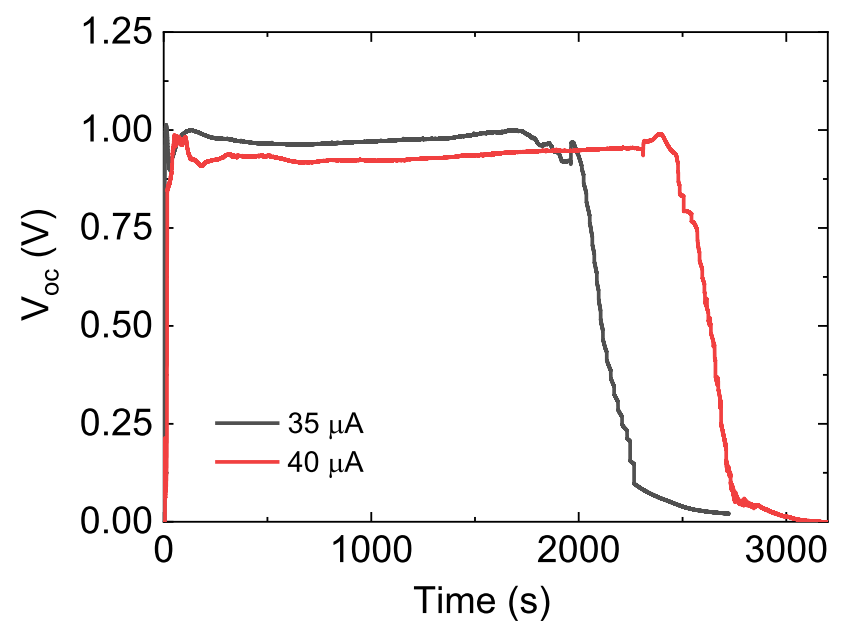


**Figure S7.** Correlation of electricity generation and volume of a water droplet. Compared to that of the 30 μL in the manuscript, the increase in volume prolonged energy generation duration but not the overall magnitude of the output.


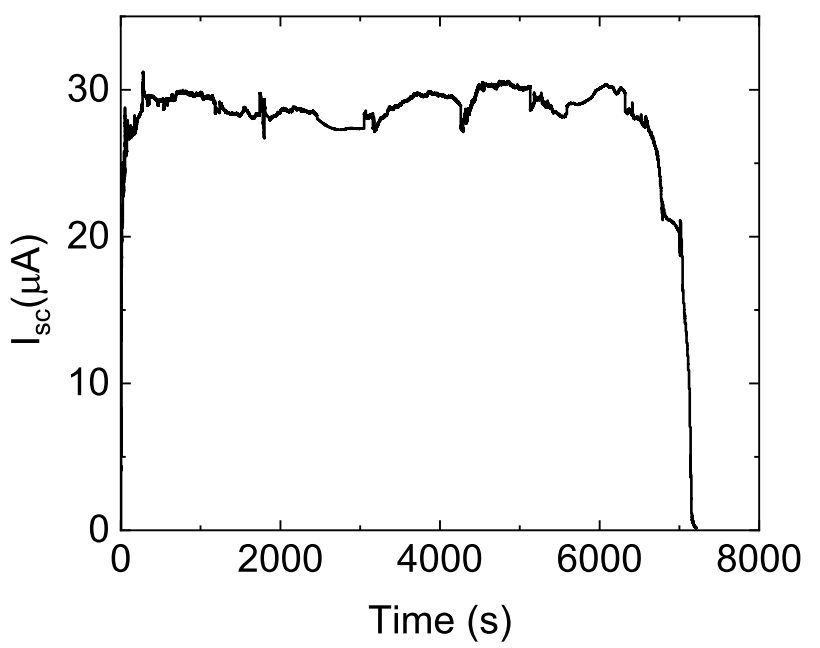

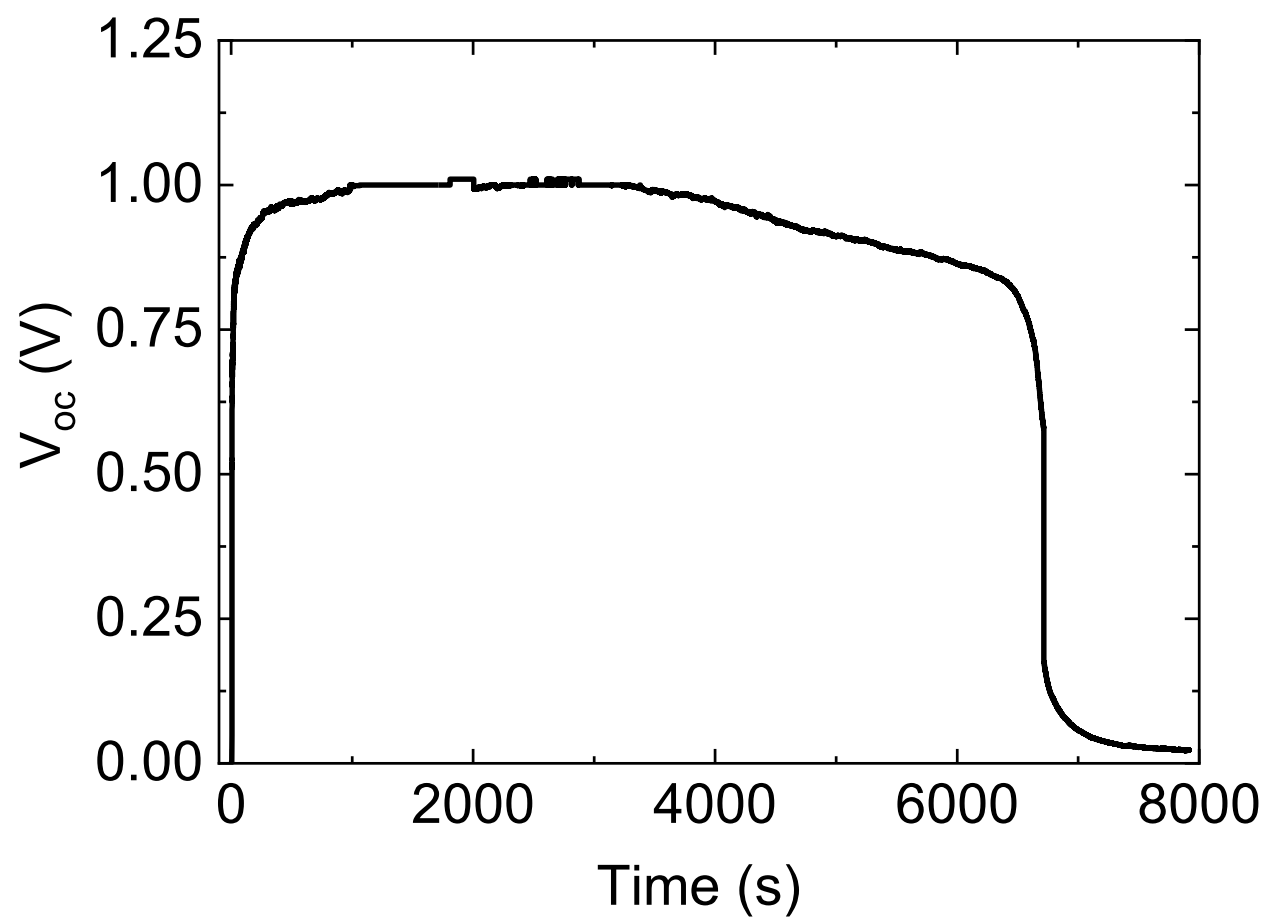


**Figure S8.** Electricity generation measurement for over 2 hours by continuously supplying water.


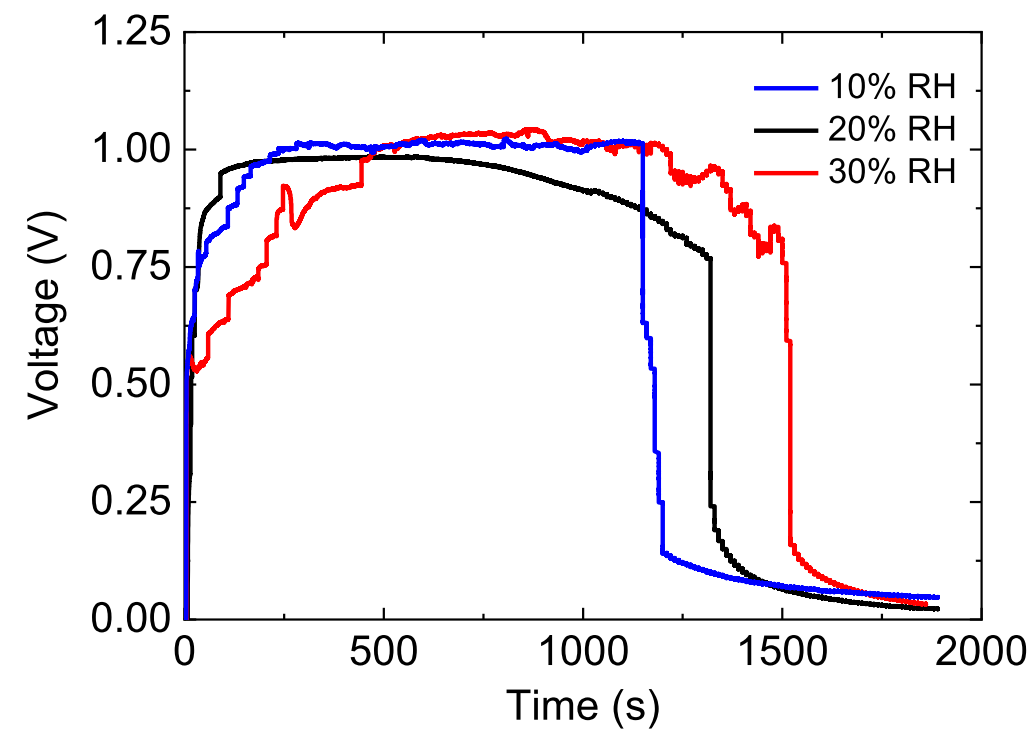


**Figure S9.** The relative humidity (RH) dependent electricity output duration. The RH at room atmosphere is around 40%, hence the variation ranges from 10 to 30% RH. It turns out that the decrease in humidity has accelerated the evaporation of a contact line which reduced the duration of electricity generation.

**Figure S10**. Temperature dependent energy generation. Generated time decreases as temperature increases.


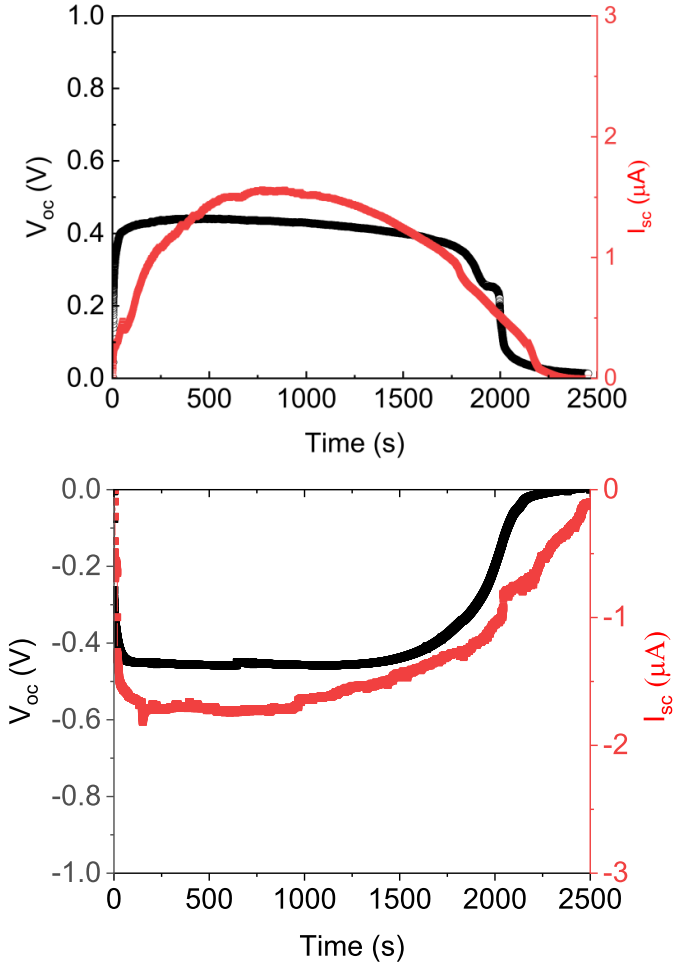


**Figure S11.** Voltage and current generation of a rGO membrane utilizing copper clips measured at each (-) and (+) side without electrode deposition. Copper clips are widely used electrode material for electrical measurement of water-induced energy harvesting. Also, the copper electrode is utilized in this study based on its almost identical work function value to that of the rGO, thus preventing the formation of potential band bending from having neither of significant ohmic nor schottky contact with rGO. *V*_oc_ and *I*_sc_ are 0.44 V and 1.56 μA, respectively.


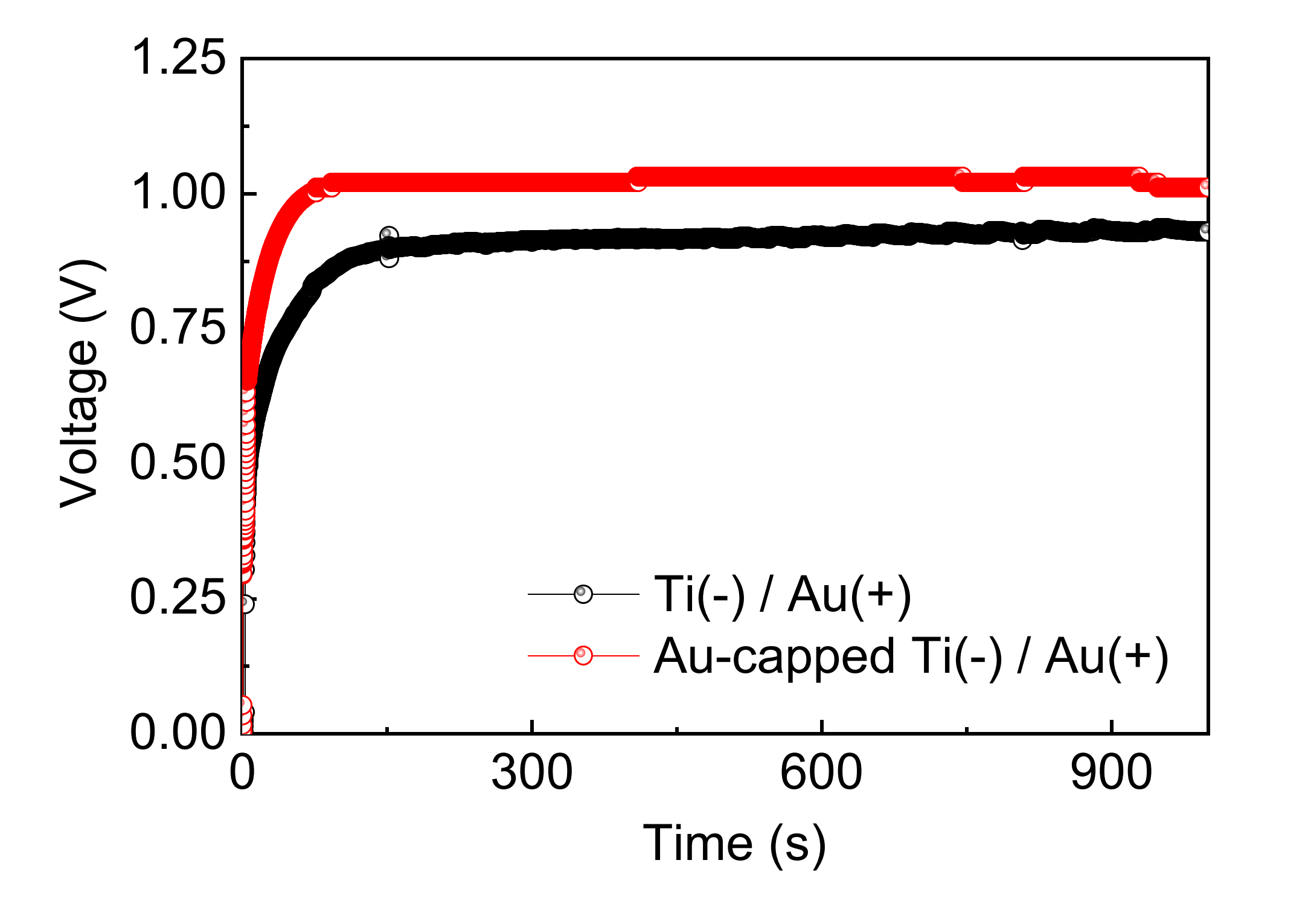


**Figure S12.** The generated electricity from Ti without the Au passivation layer. Au-capped Ti electrode (-) exhibits a little higher and more stable generation of electricity compared to that of Ti (-).


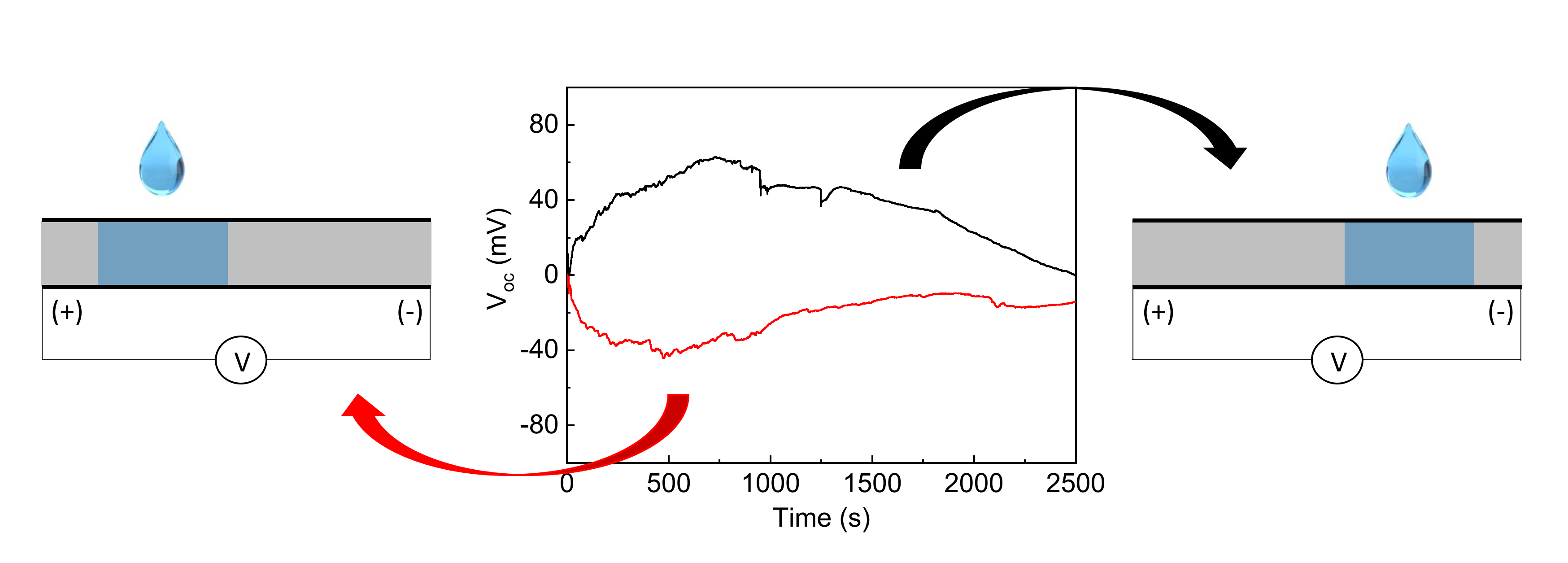


**Figure S13**. The schematic images and electrical energy generation measurements obtained by dropping a water droplet at a slightly distant position from each end of the electrodes. When water comes into contact with the rGO surface, carrier density modulation occurs, leading to potential asymmetry across the rGO. Charge carriers at the wet region would seek to flow through the external circuit via the electrodes at each near end to balance the asymmetry. However, due to the high resistance of the rGO membrane, the flow of carriers are impeded which results in the manifestation of low energy output.


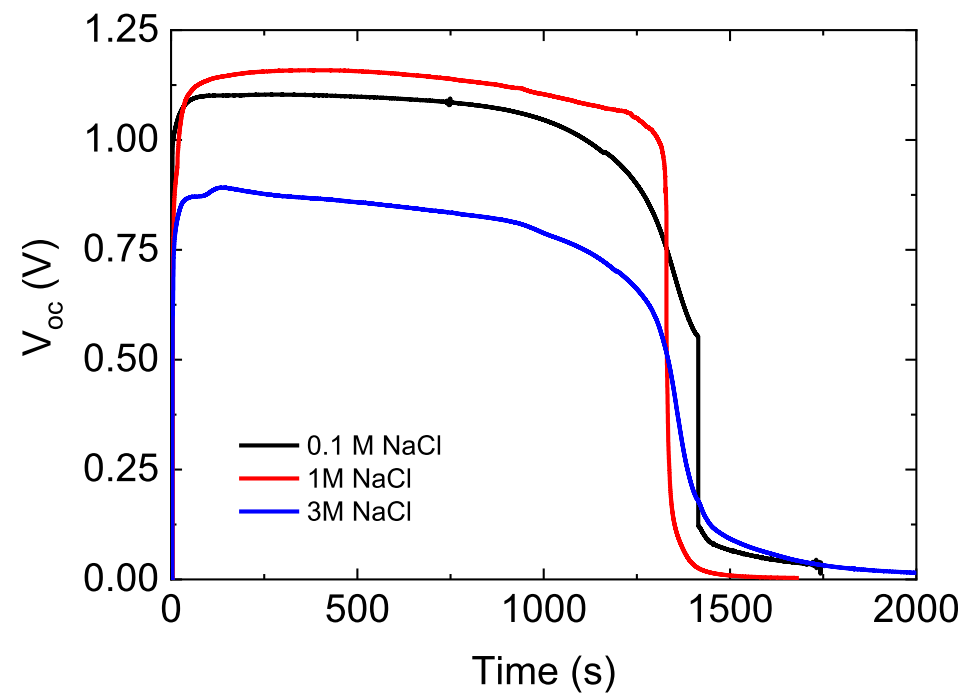


**Figure S14.** Varying concentration of the NaCl solution utilized in energy harvesting. The decrease in output performance at 3M NaCl is caused by the screening effect of counter-ions that decreases the Debye length.

**
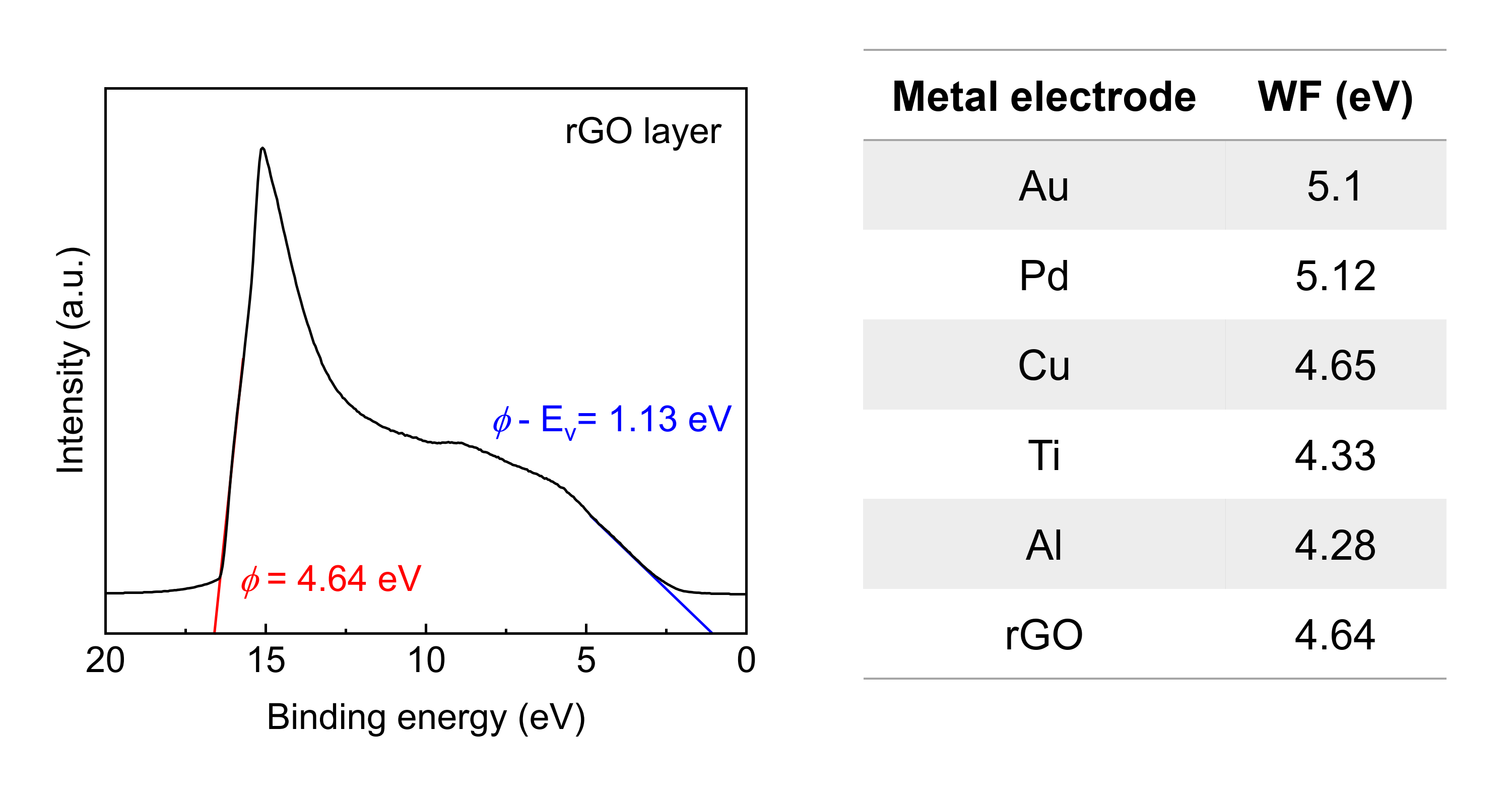
**

**Figure S15.** The work function (Ø) is measured with UPS analysis. The work function value of 4.64 eV is obtained for rGO. The table on the right shows the work function value of each metal electrode.^[4]^ Au and Pd have ohmic contact with rGO, whereas Ti and Al have schottky contact. A copper (Cu) electrode is used for measuring the electrical properties of rGO without electrode deposition.

**
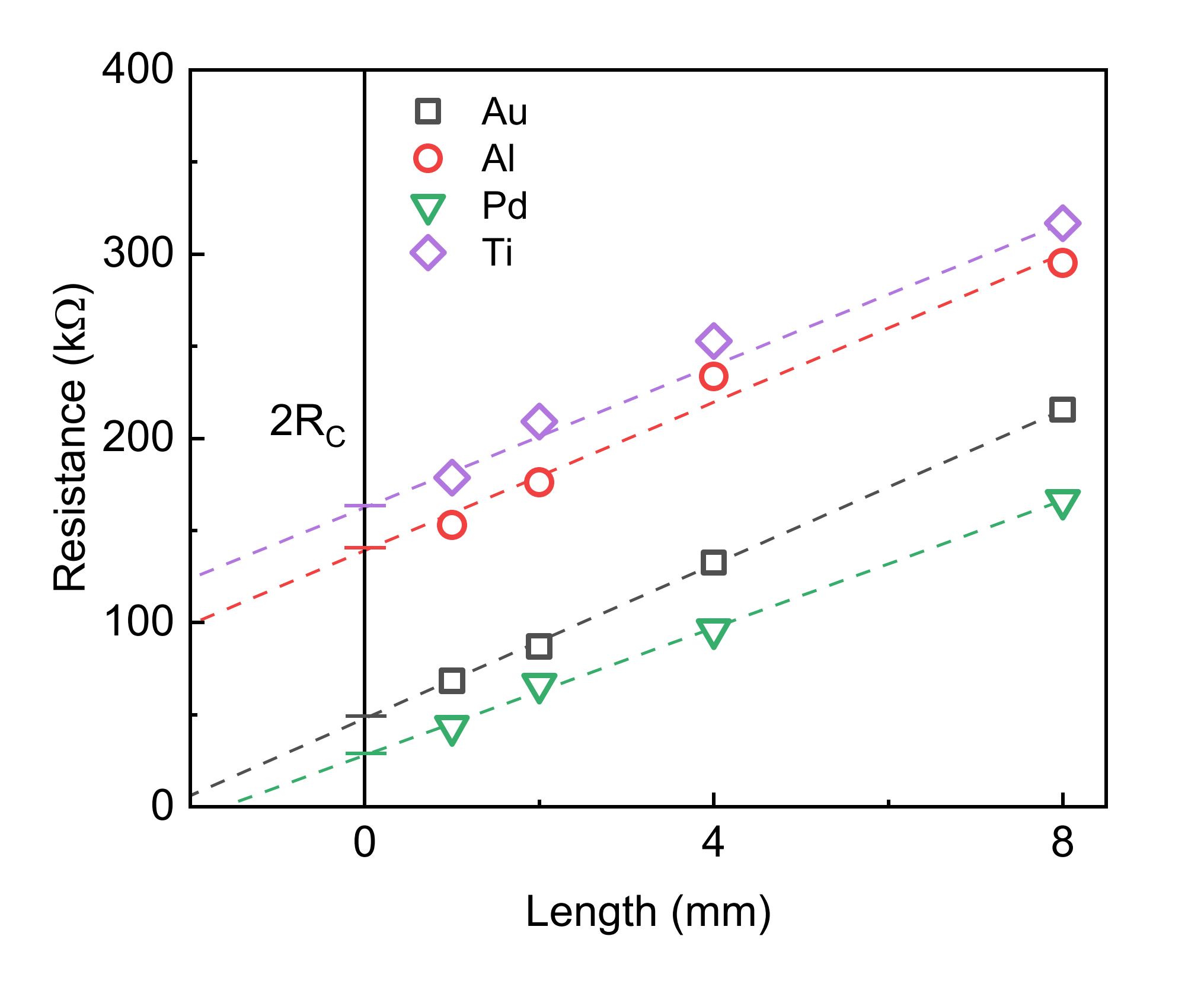
**

**Figure S16.** Calculation of contact resistance in each metal electrode by using transmission line method (TLM).

The transmission line method (TLM) was introduced to extract the contact resistance with variation of distance between each metal electrode. We measured the resistance with different distance of 1 mm, 2 mm, 4 mm, and 8 mm. Here, the total resistance can be expressed as follows,^[5]^

$R_{T}=2R_{C}+R_{B}$ (S4)

where $R_{T}$ is the total resistance, $R_{C}$ is the contact resistance between metal and rGO, and $R_{B}$ is the bulk resistance of rGO. Therefore, the total resistance is expressed in the summation of contact resistance and bulk resistance. As shown in Figure. S16, total resistance is fitted by linear plot in each contacted metal electrode, and y-intercept means double of the contact resistance. We calculated that the contact resistances of Ti, Al, Au, and Pd are 83.5 kΩ, 70.5 kΩ, 24.9 kΩ, and 15.0 kΩ, respectively. Importantly, Ti and Al form Schottky contact with rGO membrane, and Au and Pd form ohmic contact with rGO. Therefore, the difference in contact resistance is significantly due to the presence or absence of barrier potential in metal-rGO junctions. To assess the impact of contact resistance under these conditions, we should investigate the differences in contact resistance between Ti and Al, and also between Au and Pd. However, there is no significant difference in the value of contact resistance between metal electrodes according to each contact type. The difference in contact resistance for each contact type is most significantly affected by the difference in physical adhesion between the rGO membrane and the each metal electrode.

**
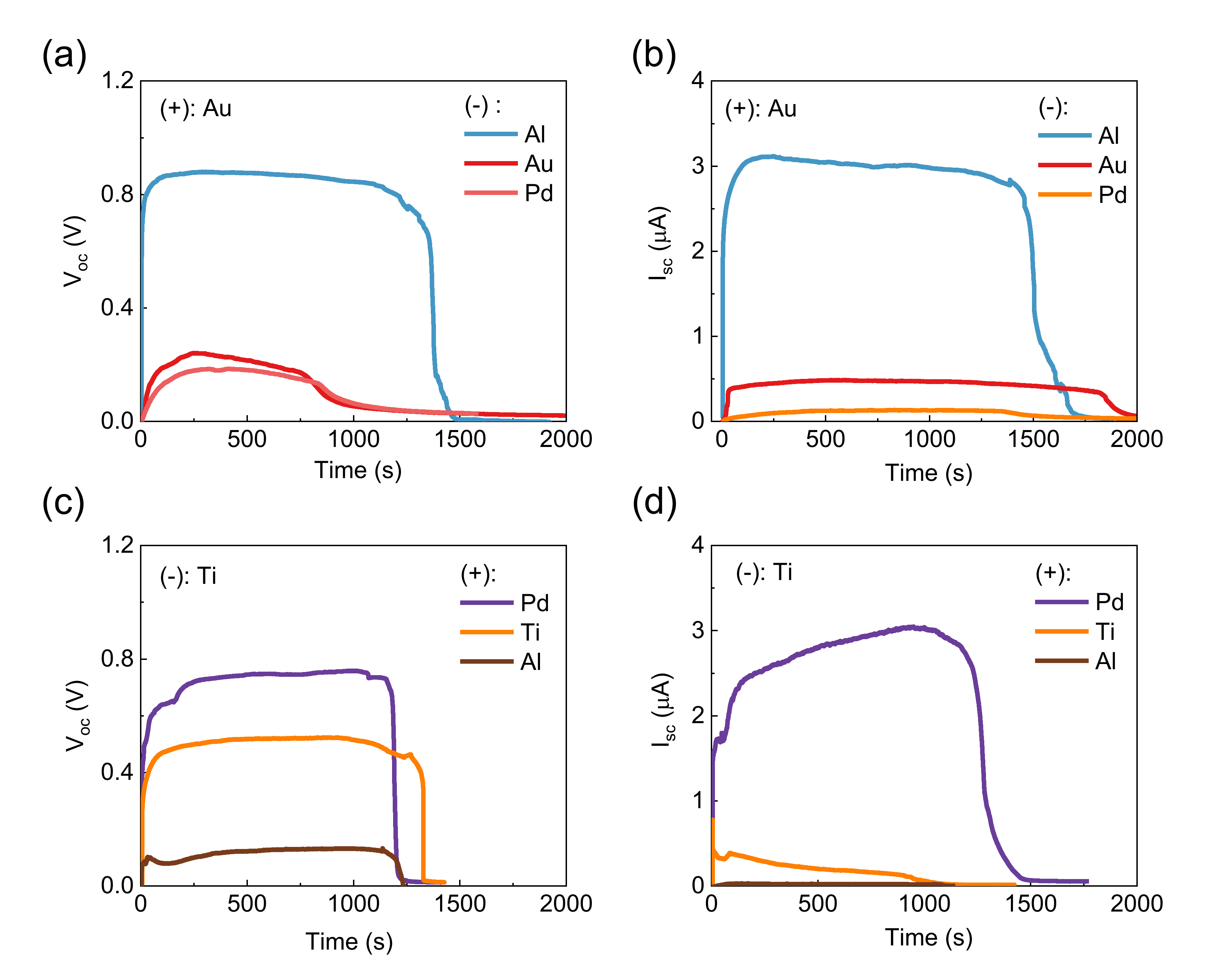
**

**Figure S17.** Electrical measurements of different electrode configurations on rGO ionovoltaic energy harvester. a-b) *V*_oc_ and *I*_sc_ of various (-) electrodes where Au is fixed as (+) electrode. c-d) *V*_oc_ and *I*_sc_ of various (+) electrodes where Ti is fixed as (-) electrode.


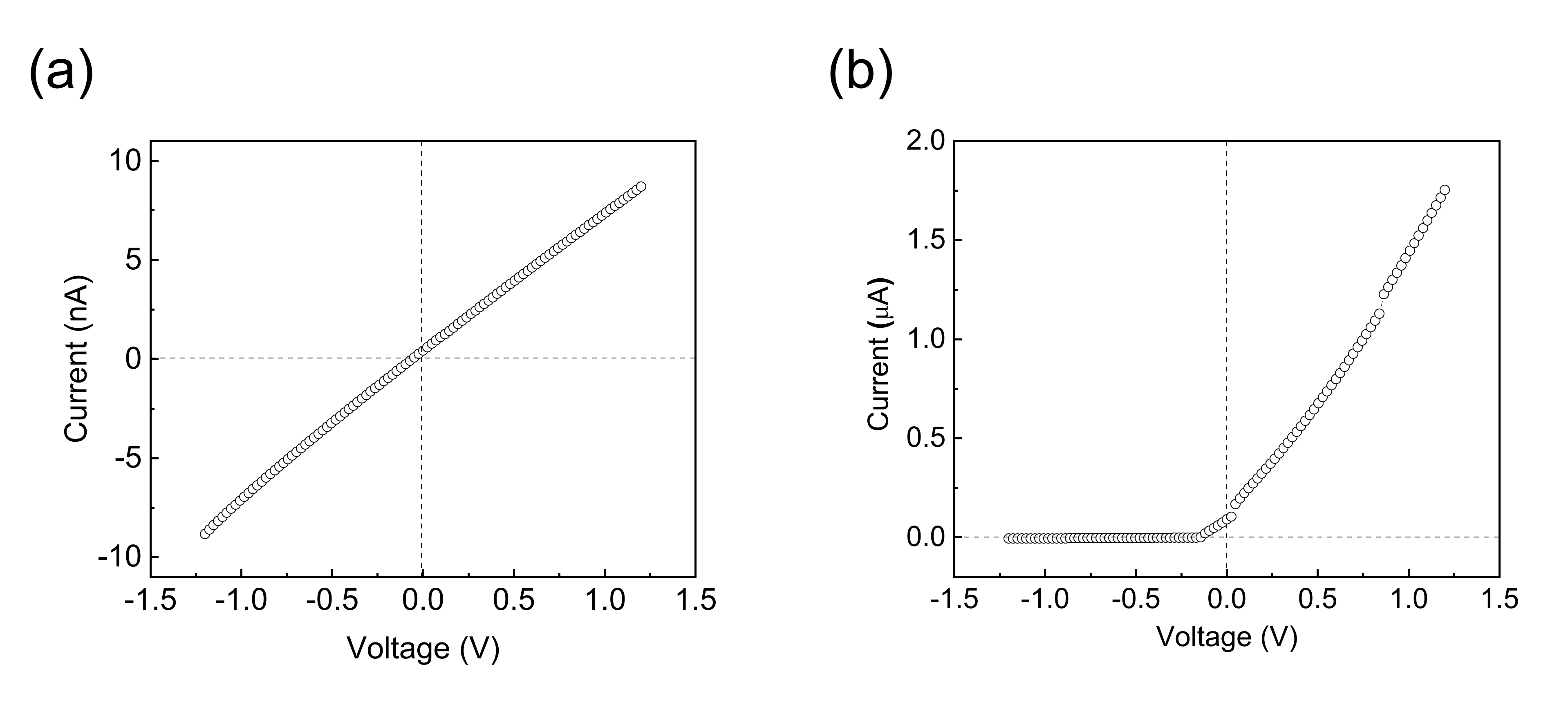


**Figure S18**. *I*-*V* curve of the pristine rGO membrane. a-b) *I*-*V* curve measured under a) the dry-state b) the wet-dry state where water is dropped on (-) electrode region.

| **I_sc_ (μA)** | **(-) electrode** | **Trial number** | | | **Average** |
| --- | --- | --- | --- | --- | --- |
|  |  | 1 | 2 | 3 |  |
|  | Al | 3.1159 | 3.6056 | 3.8645 | 3.5287 |
|  | Ti | 27.1141 | 30.8001 | 31.6267 | 29.847 |
|  | Au | 0.48517 | 0.39987 | 0.51159 | 0.46554 |
|  | Pd | 0.13407 | 0.08275 | 0.18333 | 0.13338 |
| **V_oc_ (V)** | **(-) electrode** | **Trial number** | | | **Average** |
|  |  | 1 | 2 | 3 |  |
|  | Al | 0.75867 | 0.81111 | 0.81704 | 0.795607 |
|  | Ti | 0.9568 | 1.04993 | 1.03149 | 1.012740 |
|  | Au | 0.37434 | 0.45178 | 0.34795 | 0.391357 |
|  | Pd | 0.21029 | 0.18529 | 0.2408 | 0.212127 |

| **I_sc_ (μA)** | **(+) electrode** | **Trial number** | | | **Average** |
| --- | --- | --- | --- | --- | --- |
|  |  | 1 | 2 | 3 |  |
|  | Al | 0.021637 | 0.026033 | 0.027953 | 0.025208 |
|  | Ti | 0.11454 | 0.44710 | 0.77780 | 0.44648 |
|  | Au | 5.0834 | 4.0555 | 5.2567 | 4.7985 |
|  | Pd | 3.2061 | 3.0477 | 3.9157 | 3.3898 |
| **V_oc_ (V)** | **(+) electrode** | **Trial number** | | | **Average** |
|  |  | 1 | 2 | 3 |  |
|  | Al | 0.18682 | 0.11848 | 0.13300 | 0.14610 |
|  | Ti | 0.53021 | 0.49071 | 0.52353 | 0.51481 |
|  | Au | 0.75108 | 0.83276 | 0.88036 | 0.8214 |
|  | Pd | 0.77520 | 0.75820 | 0.76453 | 0.76598 |

**Table S1**. Every *V*_oc_ and *I*_sc_ output depends on electrode configurations. Au electrode is fixed as (+) electrode for the table at the top. The Ti electrode is fixed as (-) electrode for the table below. Electrical measurements are conducted three times for each configuration to obtain the average value.

| **Publication year** | **Material** | **Supply method** | **Power density (µW/cm^2^)** | **Reference** |
| --- | --- | --- | --- | --- |
| 2019 | Al_2_O_3_ | Evaporation | 0.0277 | ^[6]^ |
| 2020 | SiNWs | Evaporation | 6 | ^[7]^ |
| 2020 | CuNWs | Droplet capillary | 0.0115 | ^[8]^ |
| 2021 | rGO | Evaporation | 0.79 | ^[9]^ |
| 2021 | SiNWs | Droplet capillary | 10 | ^[10]^ |
| 2021 | MoS_2_ | Evaporation | 0.0185 | ^[11]^ |
| 2022 | MoS_2_ | Evaporation | 0.335 | ^[12]^ |
| 2022 | Titanium carbide/Mxene | Evaporation | 8.228 | ^[13]^ |
| 2022 | Carbon black | Evaporation | 0.05446 | ^[14]^ |
| 2023 | Graphene | Droplet capillary | 0.0264 | ^[15]^ |
| 2023 | Functionalized graphite | Evaporation | 0.035 | ^[16]^ |
| 2024 | rGO/polymer | Evaporation | 0.3456 | ^[17]^ |
| **This work** | **rGO** | **Droplet capillary** | **16.59** |  |

**Table S2.** Power density output comparison with recently reported water-induced energy harvesters. Every measurement has a continuous generation of energy from the relative motion at the solid-liquid interface with deionized water.

**Supporting References**

[1] M. Lundie, Ž. Šljivančanin, S. Tomić, *J. Mater. Chem. C* **2015**, *3*, 7632.

[2] T. T. Pham, T. N. Pham, V. Chihaia, Q. A. Vu, T. T. Trinh, T. T. Pham, L. Van Thang, D. N. Son, *RSC Adv.* **2021**, *11*, 19560.

[3] W.-C. Peng, Y.-C. Chen, J.-L. He, S.-L. Ou, R.-H. Horng, D.-S. Wuu, *Sci. Rep.* **2018**, *8*, 9255.

[4] B. Ofuonye, J. Lee, M. Yan, C. Sun, J.-M. Zuo, I. Adesida, *Semicond. Sci. Technol.* **2014**, *29*, 95005.

[5] C. Im, J. Kim, N.-K. Cho, J. Park, E. G. Lee, S.-E. Lee, H.-J. Na, Y. J. Gong, Y. S. Kim, *ACS Appl. Mater. Interfaces* **2021**, *13*, 51266.

[6] C. Shao, B. Ji, T. Xu, J. Gao, X. Gao, Y. Xiao, Y. Zhao, N. Chen, L. Jiang, L. Qu, *ACS Appl. Mater. Interfaces* **2019**, *11*, 30927.

[7] Y. Qin, Y. Wang, X. Sun, Y. Li, H. Xu, Y. Tan, Y. Li, T. Song, B. Sun, *Angew. Chemie - Int. Ed.* **2020**, *59*, 10619.

[8] H. Jin, S. G. Yoon, W. H. Lee, Y. H. Cho, J. Han, J. Park, Y. S. Kim, *Energy Environ. Sci.* **2020**, *13*, 3432.

[9] S. G. Yoon, H. Jin, W. H. Lee, J. Han, Y. H. Cho, Y. S. Kim, *Nano Energy* **2021**, *80*, 105522.

[10] B. Shao, Z. Song, X. Chen, Y. Wu, Y. Li, C. Song, F. Yang, T. Song, Y. Wang, S. T. Lee, B. Sun, *ACS Nano* **2021**, *15*, 7472.

[11] M. Wu, M. Peng, Z. Liang, Y. Liu, B. Zhao, D. Li, Y. Wang, J. Zhang, Y. Sun, L. Jiang, *ACS Appl. Mater. Interfaces* **2021**, *13*, 26989.

[12] Z. Guo, J. Wang, Y. Wang, J. Wang, J. Li, T. Mei, J. Qian, X. Wang, *Chem. Eng. J.* **2022**, *427*, 131008.

[13] J. Bae, M. S. Kim, T. Oh, B. L. Suh, T. G. Yun, S. Lee, K. Hur, Y. Gogotsi, C. M. Koo, I. D. Kim, *Energy Environ. Sci.* **2022**, *15*, 123.

[14] S. Fang, J. Li, Y. Xu, C. Shen, W. Guo, *Joule* **2022**, *6*, 690.

[15] H. Kong, H. Yao, Y. Li, Q. Wang, X. Qiu, J. Yan, J. Zhu, Y. Wang, *ACS Nano* **2023**, *17*, 18456.

[16] R. Kumar, G. Kay, G. Beaton, G. Liu, K. Stamplecoskie, *ACS Appl. Mater. Interfaces* **2023**, *15*, 7511.

[17] S. Yu, Y. H. Cho, W. H. Lee, S. G. Yoon, J. Park, J. Han, L. Li, H. Jin, Y. S. Kim, *Nano Energy* **2024**, *123*, 109345.
